# Barbier Self-Condensing Ketyl Polymerization-Induced Emission: A Polarity Reversal Approach to Reversed Polymerizability

**DOI:** 10.1016/j.isci.2020.101031

**Published:** 2020-04-05

**Authors:** Shun-Shun Li, Nengbo Zhu, Ya-Nan Jing, Yajun Li, Hongli Bao, Wen-Ming Wan

**Affiliations:** 1 State Key Laboratory of Structural Chemistry, Key Laboratory of Coal to Ethylene Glycol and Its Related Technology, Center for Excellence in Molecular Synthesis, Fujian Institute of Research on the Structure of Matter, Chinese Academy of Sciences, 155 West Yangqiao Road, Fuzhou 350002, P. R. of China; 2 University of Chinese Academy of Sciences, Beijing 100049, P. R. of China; 3 State Key Laboratory of Heavy Oil Processing and Center for Bioengineering and Biotechnology, China University of Petroleum (East China), 66 West Changjiang Road, Qingdao 266580, P. R. of China

**Keywords:** Organic Chemistry, Optical Property, Polymers

## Abstract

Carbon-carbon bond formation through polarity reversal ketyl radical anion coupling of carbonyls has inspired new reaction modes to this cornerstone carbonyl group and played significant roles in organic chemistry. The introduction of this resplendent polarity reversal ketyl strategy into polymer chemistry will inspire new polymerization mode with unpredicted discoveries. Here we show the successful introduction of polarity reversal ketyl approach to polymer chemistry to realize self-condensing ketyl polymerization with polymerization-induced emission. In this polarity reversal approach, it exhibits intriguing reversed polymerizability, where traditional excellent leaving groups are not suitable for polymerization but challenging polymerizations involving the cleavage of challenging C-F and C-CF_3_ bonds are realized under mild Barbier conditions. This polarity reversal approach enables the polymer chemistry with polarity reversal ketyl mode, opens up a new avenue toward the polymerization of challenging C-X bonds under mild conditions, and sparks design inspiration of new reaction, polymerization, and functional polymer.

## Highlights

• Self-condensing ketyl polymerization
• Polymerization-induced emission
• C-F/C-CF_3_ activation polymerization
• Polarity reversal and reversed polymerizability

## Graphical Abstract

**Figure:**
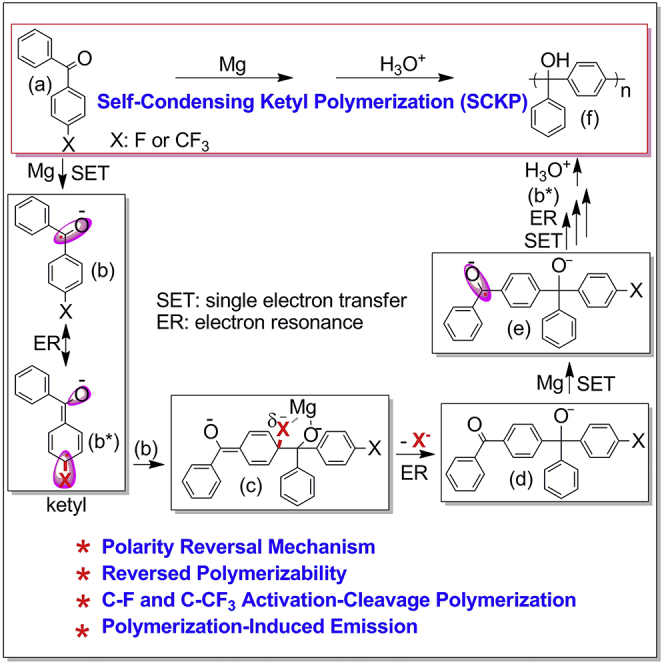

## Introduction

Carbon-carbon (C-C) bond formation plays a central role in modern organic synthesis. In comparison with C-C bond formation through classic polar coupling mechanism, C-C bond formation through polarity reversal ketyl radical anion (ketyl) coupling of carbonyls enables access to a polarity-reversed platform with reactivity umpolung and has inspired new reaction modes to this cornerstone carbonyl group in organic chemistry (Hart, 1984, Wang et al., 2017, Wang et al., 2018). The development of new polymerization methodology based on this resplendent polarity reversal ketyl strategy will inspire new polymerization mode with unpredicted discoveries, expand the structure and functionality libraries of monomer and polymer, and open up a new avenue for the design and application of polymer materials.

**Figure 1:**
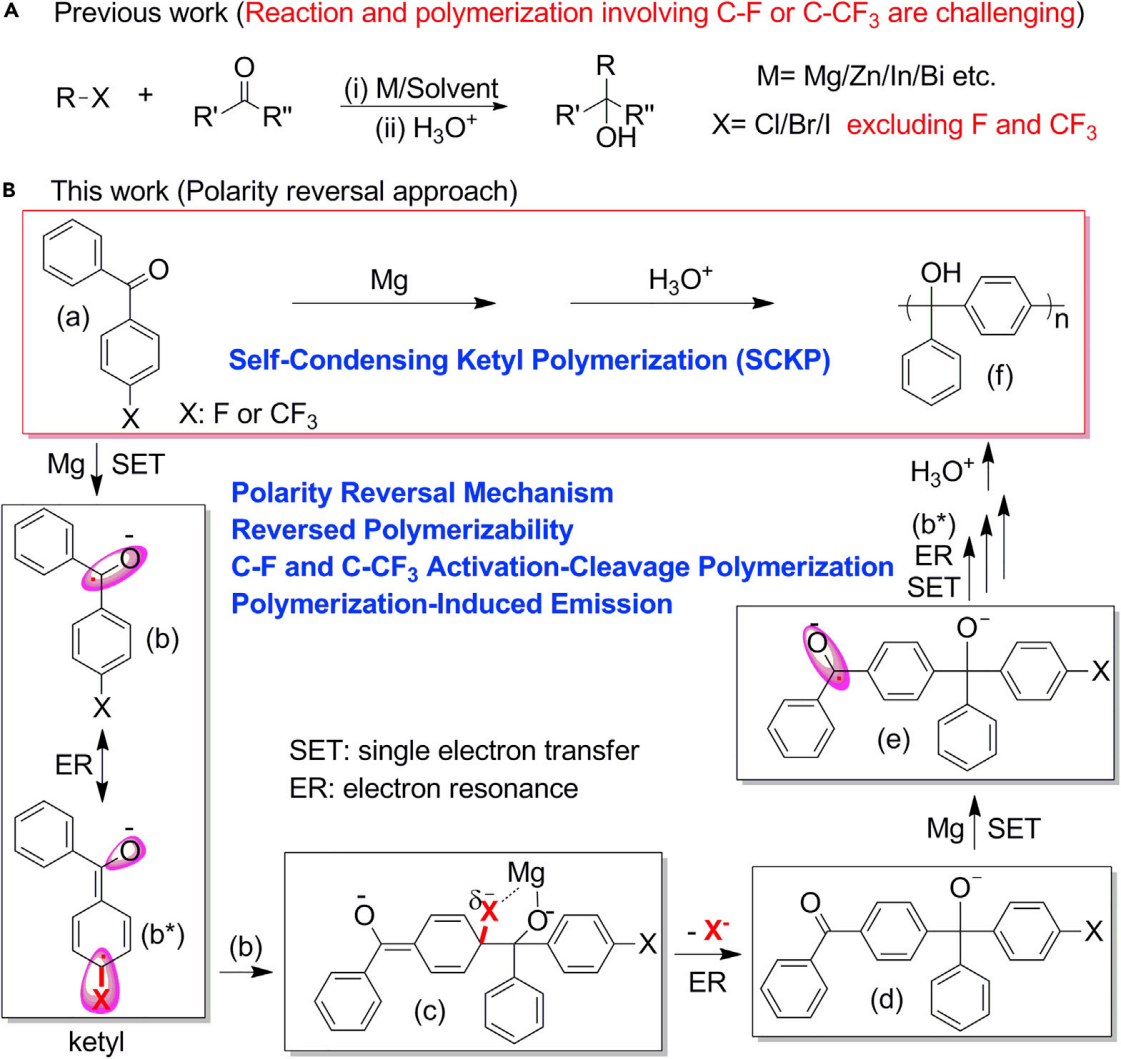
The Introduction of Polarity Reversal Ketyl Strategy into Polymer Chemistry The introduction of polarity reversal ketyl strategy into polymer chemistry to realize self-condensing ketyl polymerization triggered by electron resonance, enabling reversed polymerizability toward C-F and C-CF_3_ activation-cleavage polymerization. (A) Previous work of the Barbier reaction involving challenging C-F or C-CF_3_. (B) Polarity reversal strategy via self-condensing ketyl polymerization in this work.

Classical C-C bond formation reactions, including atom transfer radical addition reaction (Pintauer and Matyjaszewski, 2008, Wang and Matyjaszewski, 1995), radical addition-fragmentation reaction (Chiefari et al., 1998, Moad et al., 2008), olefin metathesis reaction (Bielawski and Grubbs, 2000, Bielawski and Grubbs, 2007, Vougioukalakis and Grubbs, 2010), Suzuki coupling reaction (Baggett et al., 2015, Kotha et al., 2002, Littke et al., 2000, Miyaura et al., 1981, Schluter, 2001, Yokoyama et al., 2007), Michael addition reaction (Hong et al., 2007, Liu et al., 2003, Wang et al., 2005), Stille coupling reaction (Bao et al., 1995, Guo et al., 2014, Littke and Fu, 1999, Yin et al., 2016), click chemistry reactions (He et al., 2016, He et al., 2017), multiple components reactions (Deng et al., 2012, Deng et al., 2016, Kreye et al., 2011, Wei et al., 2017, Wu et al., 2017, Xue et al., 2016), the Barbier reaction (Jing et al., 2019, Sun et al., 2017), and radical cascade reaction (Zhu et al., 2020), have been introduced to polymer chemistry to develop polymerization methodologies (Hawker and Wooley, 2005, Huang et al., 2019, Iha et al., 2009, Jiang et al., 2018, Song et al., 2018, Tsao and Wooley, 2017). Generally, C-C bond formation is initiated by activation-cleavage of C-X bonds, such as C-I, C-Br, C-Cl, C-S, C-O, and C-B, and these C-X bond activation-cleavage polymerizations have been well established in polymer chemistry with X as traditional leaving group. In comparison, both organic reaction and polymerization involving C-F or C-CF_3_ bond activation-cleavage have not been well realized yet and are significantly challenging, taking Barbier reaction as an example (Figure 1A), (Berger et al., 2018, Blomberg, 1993, Blomberg and Hartog, 1977, Li and Zhang, 1998, Sergeev et al., 1982, Zhang and Li, 1999, Zhou and Li, 2014) stemming from the extremely high bond dissociation energy (Ahrens et al., 2015, Amii and Uneyama, 2009, Cui et al., 2018, Meissner et al., 2017, Tian et al., 2015).

Owing to the high bond dissociation energy of the C-F and C-CF_3_ bonds, activation-cleavage of these bonds through direct insertion of Mg into the C-F or C-CF_3_ bond is difficult. Taking C-F activation-cleavage in Barbier reaction as an example, we hypothesize that, instead, Mg will react with the carbonyl group in the formation of ketyl through a reductive polarity reversal mechanism via single electron transfer (SET) and Mg will interact with C-F bond through van der Waals forces as well, resulting in nucleophilic addition of a ketyl to the C-F bond and formation of a five-membered ring intermediate (Figure S1). Mg-mediated C-F activation-cleavage and addition to a carbonyl group can therefore be realized in a one-pot Barbier reaction through polarity reversal ketyl mechanism. Furthermore, in the case of 4-fluorobenzophenone ([a] in Figure 1B), a SET between Mg and 4-fluorobenzophenone (a) will produce a ketyl (b) through reductive polarity reversal mechanism, where (b∗) is another electron resonance (ER) structure of ketyl of (b). Self-condensing coupling between (b) and (b∗) will initiate the self-condensing dimerization of ketyl, which involves formation of (c), cleavage of the C-F bond and ER in the formation of a carbonyl end group (d), and a SET between (d) and Mg forming a ketyl dimer (e). Continuous chain propagation with (b∗) as monomer followed by ER and SET processes, Mg-mediated C-F activation-cleavage polymerization can be realized by polarity reversal strategy through self-condensing ketyl polymerization (SCKP) of (a), similar to the self-condensing concept of self-condensing vinyl polymerization proposed by Fréchet (Fréchet et al., 1995, Hawker et al., 1995, Liu et al., 1999).

Herein, we demonstrate the successful introduction of polarity reversal ketyl strategy to polymer chemistry to realize SCKP. Through this polarity reversal SCKP, the polymerizability of monomers gets reversed, where traditional excellent leaving groups are not suitable for polymerization but challenging polymerizations involving the cleavage of challenging C-F and C-CF_3_ bonds are realized under mild Barbier conditions. This new polymerization mode also exhibits intriguing tunable polymerization-induced emission properties by simply adjusting monomer structure and polymerization time. This work therefore enables the polymer chemistry with polarity reversal ketyl mode with reversed polymerizability and opens up a reversed and feasible strategy for the polymerization of challenging monomers, which might inspire new reaction, polymerization, and luminescent polymer design.

## Results and Discussion

**Figure 2:**
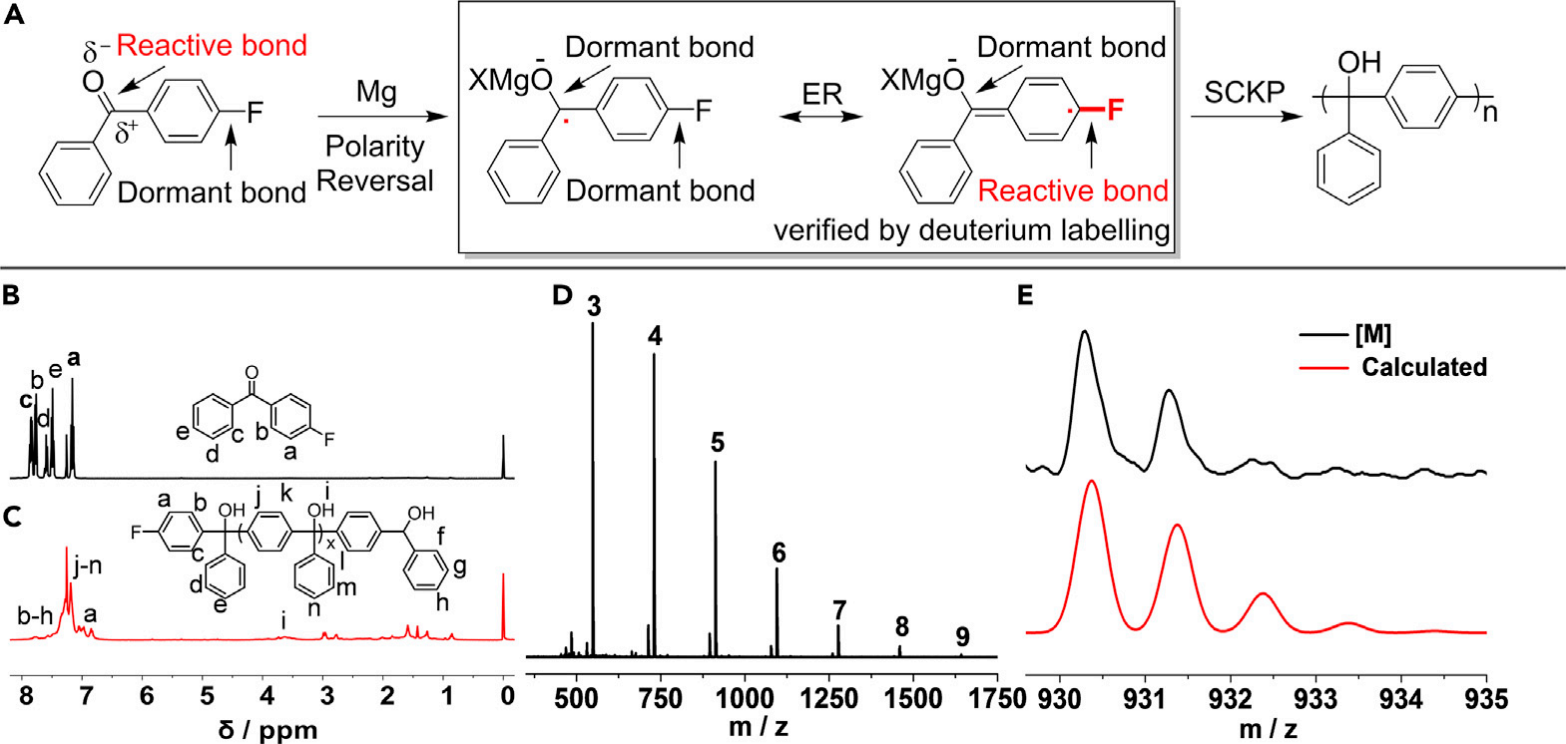
Process of Self-Condensing Ketyl Polymerization and Characterizations of Fluoro-poly(triphenylmethanol) (fluoro-PTPM) Synthesized with 4-fluorobenzophenone as Monomer (A) Proposed mechanism of self-condensing ketyl polymerization. (B) ^1^H NMR spectrum of 4-fluorobenzophenone in CDCl_3_. (C) ^1^H NMR spectrum of fluoro-PTPM in CDCl_3_. (D) Complete MALDI-TOF spectrum of fluoro-PTPM. (E) Comparison between observed and calculated MALDI-TOF mass spectra of pentamer [M].

To demonstrate the above hypothesis of reductive polarity reversal mechanism, the reaction between fluorobenzene and carbonyl compounds was carried out under Barbier conditions, where the reaction exhibits reversed reactivity. The reactivity order of aromatic halides in the polarity reversal ketyl mechanism is C-F > C-Cl (Figure S1 and Tables S1–S3), whereas the conventional reactivity order of aromatic halides is C-Cl >> C-F. Such reaction involving Mg and fluorobenzene is thought of as sluggish, and this result therefore confirms the significance of polarity reversal ketyl mechanism and is therefore of significant importance in organic chemistry. To further confirm the hypothesis of SCKP by introducing polarity reversal ketyl strategy into polymer chemistry, the polymerization of 4-fluorobenzophenone as monomer was carried out in the presence of Mg (1.2 eq.) and 1, 2-dibromoethane (0.2 eq.) at 45°C in THF for 24 h (Figure 4 entry 1). As shown in Figure 2A, the C-F bond is a dormant bond before polarity reversal of reactive C=O bond. After reductive polarity reversal of C=O bond in the formation of ketyl, followed by ER, the dormant C-F bond becomes reactive bond. The activation-cleavage of challenging C-F bond is therefore realized in the polarity reversal strategy, which further enables the SCKP of challenging monomer containing C-F bond.

From the characterizations of the products shown in Figures 2 and S3, the successful formation of polymer can be verified obviously from the comparison of NMR spectra, where sharp ^1^H NMR signals of monomer disappear and broader aromatic and hydroxide signals of polymer appear at 7.60–6.75 and 3.83–3.35 ppm, respectively. From comparison of the ^13^C NMR spectra of the monomer and the polymer, the carbonyl signal at 195.33 ppm can be seen to disappear and the fluorobenzene signals at 166.67 and 164.15 ppm decrease, which confirms that the polymer contains fluorobenzene as an end group. The existence of this fluorobenzene end group is confirmed by ^19^F NMR signal at 115.46 ppm. The successful formation of the polymer is further verified by gel permeation chromatography (GPC) characterization with a *M*_n_ of 3,400 g/mol and *Ð* of 1.40. The chemical structure of the polymer is further verified by the Fourier transform infrared (FTIR) spectrum, which contains groups like C-OH, phenyl, and fluorobenzene. The characteristic glass transition temperature (Tg) of fluoro-poly(triphenylmethanol) (fluoro-PTPM) was measured by differential scanning calorimetry (DSC) with a Tg of ~104.7°C (Figure S3).

To confirm the polymerization mechanism, matrix-assisted laser desorption ionization time-of-flight (MALDI-TOF) mass spectrometry was utilized to characterize the polymer sample obtained after 10 min of polymerization, as shown in Figures 2D–2F and S4. From the complete spectrum, an m/z difference of 182.196 can be clearly observed as the molecular weight of the repeating unit, which is consistent with the molecular weight of diphenylmethanol group (182.073 Da). From the enlarged spectrum of pentamer, every peak can be identified and assigned as [M]-3OH, [M]-2OH, [M]-OH-nH, [M], and [M]+Na^+^. The chemical structure of fluoro-PTPM is further supported by comparison of the similarity between the observed and calculated isotope peaks of [M], [M]-OH-nH, and [M]-2OH, signals that show a very similar isotope pattern. All these results confirm the hypothesized polarity reversal SCKP mechanism shown in Figures 1B and 2A, which relies on the polarity reversal of reactive ketone group in the formation of ketyl intermediates and ER triggered C-F bond activation-cleavage, resulting in polymerization through further SCKP.

**Figure 3:**
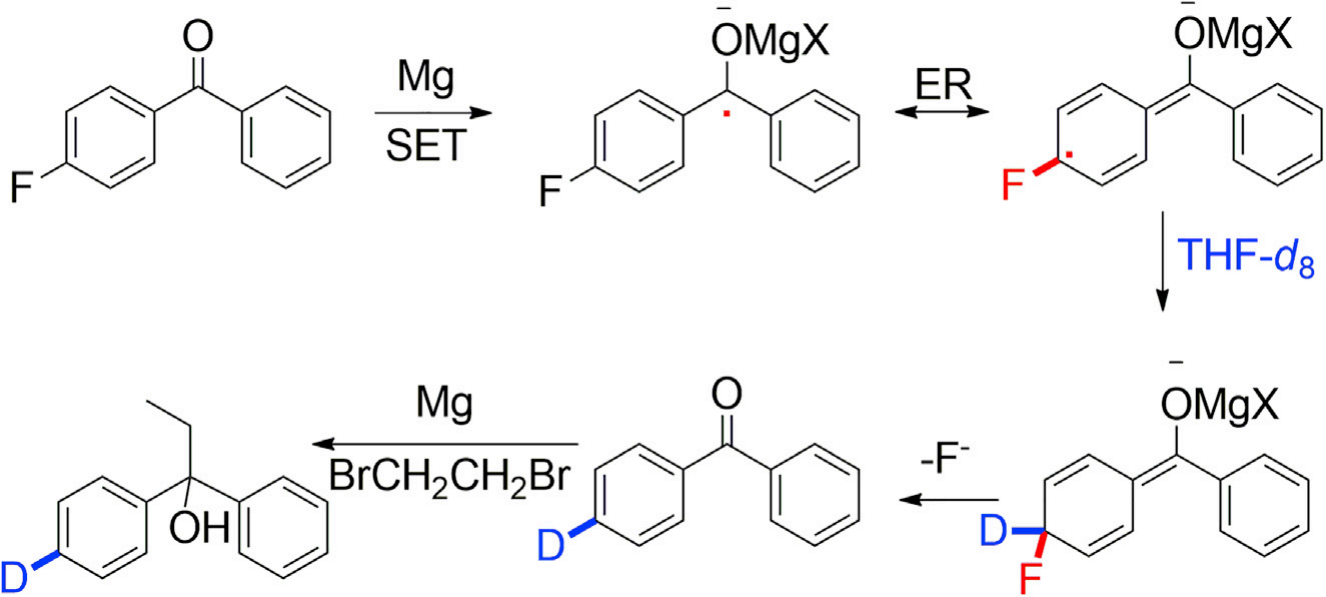
Validation of the Self-Condensing Ketyl Polymerization Mechanism via a Deuterium Labeling Experiment

**Figure 4:**
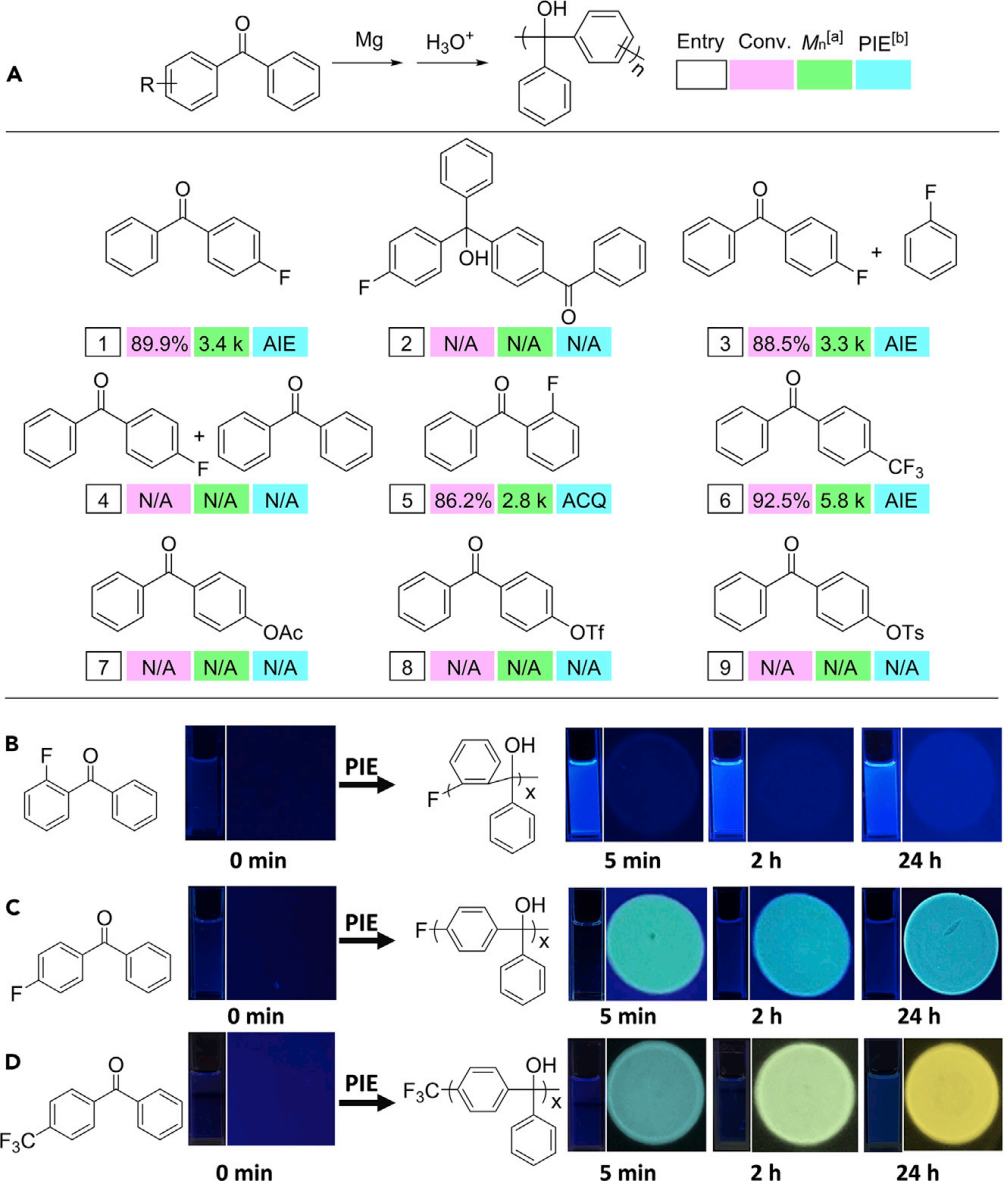
Results of Self-Condensing Ketyl Polymerization Reaction conditions: monomer (1.0 g, 1.0 eq.), Mg (1.2 eq.), 1, 2-dibromoethane (0.2 eq.), THF (10 mL), 45°C, 24 h; ^[a]^measured by GPC; ^[b]^polymerization-induced emission. (A) Substrate range of self-condensing ketyl polymerization. (B) Emission digital photos of polymerization process of 2-fluorobenzophenone at different polymerization times (under irradiation with UV lamp at 365 nm). (C) Emission digital photos of polymerization process of 4-fluorobenzophenone at different polymerization times (under irradiation with UV lamp at 365 nm). (D) Emission digital photos of polymerization process of 4-trifluoromethylbenzophenone at different polymerization times (under irradiation with UV lamp at 365 nm).

To further confirm the above polymerization mechanism, a deuterium labeling experiment was carried out in THF-*d*_8_ with 3 eq. of Mg and 1 eq. of 1, 2-dibromoethane at 45°C. The greatly increased amounts of Mg and 1, 2-dibromoethane will influence the reductive polarity reversal process via SET and interrupt the polymerization process, so that the intermediate should be captured. Through the deuterium labeling experiment, *para*-deuterated diphenylethylmethanol with 6% isolated yield and 35% deuterated ratio was successfully captured (Figure 3 and Figures S28–S32). According to the analysis shown in Figure 3, the only possible pathway to the formation of *para*-deuterated diphenylethylmethanol requires the reductive polarity reversal of carbonyl via a SET in the formation of a ketyl radical anion intermediate, the C-F activation via ER of the ketyl radical anion intermediate, the hydrogen/deuterium abstraction reaction between the ER ketyl radical anion intermediate and THF-*d*_8_ as found in the literature (Clerici et al., 2005, Pryor et al., 1983), the C-F cleavage via ER in the formation of carbonyl compound, and the addition of the Grignard reagent. To further clarify the polymerization process of SCKP through polarity reversal ketyl mechanism, ^1^H NMR and FTIR techniques were carried out with different substituents as leaving groups (Figures S8–S12 and 4A). The failure of polymerization of AB-type dimer containing both C-F and C=O moieties indicates the importance of C-F activation-cleavage triggered by ER in SCKP (Figure 4A entry 2). This also indicates that the polymerization mechanism of SCKP is not a traditional AB-type polycondensation, which is further confirmed by controlled experiments of polymerization of AB-type bifunctional 4-fluorobenzophenone in the presence of 1 eq. of monofunctional fluorobenzene or benzophenone. In this nontraditional AB-type polycondensation, monofunctional fluorobenzene did not inhibit the polymerization but monofunctional benzophenone did (Figure 4A entries 3 and 4). The evidences of obvious polymer signal observed at 15 min with conversion as low as 28.8%, successful isolation of 3.2% yield of dimer (d) after 24 h of polymerization, and still more than 10% of monomer observed after 24 h of polymerization also confirm this SCKP is not a traditional AB-type polycondensation (Figures S5 and S6). FTIR tracing experiments verify that this SCKP process contains carbonyl addition process and C-F activation-cleavage process with decreased carbonyl signal, increased C-O signal, and decreased fluorophenyl signal (Figure S6).

These above results therefore confirm the proposed polymerization mechanism shown in Figure 1B. In the reductive polarity reversal mechanism, carbonyl addition happens first via a SET between carbonyl and Mg in the formation of ketyl. C-F activation happens second via ER of ketyl and, third, is followed by a coupling reaction between (b) and (b∗) to initiate the polymerization of (b∗). The fourth step is the C-F cleavage via ER in the formation of the carbonyl end group, which will further react with Mg via SET to complete chain propagation with one monomer inserted. Attributed to the continuous relay of reactions in the order of carbonyl addition via SET in the formation of ketyl, C-F activation via ER, and ketyl coupling and C-F cleavage via ER, the SCKP is realized through polarity reversal ketyl mechanism. This polarity reversal ketyl mechanism exhibits intriguing reversed polymerizability as well. With traditional leaving groups like acetoxy (OAc), tosylate (OTs), and triflate (OTf), which could react with Mg directly, their failures in polymerization indicate that the direct activation-cleavage of leaving groups by Mg would interrupt SCKP (Figure 4A entry 7–9). However, with even more challenging trifluoromethyl (CF_3_) substituent as leaving group, its SCKP is realized successfully (Figure 4A entry 6). These results indicate that polarity reversal strategy of SCKP enables the polymerization of challenging monomers through activation-cleavage of challenging bonds under mild conditions. It is worth mentioning that this discovery might open up a reversed and feasible strategy for the polymerization of challenging monomers, which might further inspire new reaction and polymerization.

Photophysical properties are intriguing phenomena with potential optical, electronic, and sensory applications (Lv et al., 2017, Lv et al., 2019, Wan et al., 2017, Wan et al., 2018). To illustrate the advantage of this SCKP, photophysical properties of the obtained PTPMs and the polymerization process were investigated (Figures 4B–4D and S13–S16). From the results, these PTPMs exhibit obviously site-specific luminescence that can be simply adjusted by monomer structures. When the phenyl rings of obtained *para*-PTPMs are rotation free, they are aggregation-induced emission (AIE)-type luminescence, similar to Tang's reports (Hong et al., 2011, Liu et al., 2020, Luo et al., 2001, Mei et al., 2015, Zhang et al., 2017). When the rotation about the phenyl rings is hindered, the *ortho*-PTPM exhibits traditional luminescence with aggregation caused quenching (ACQ) effect. These results confirm that PTPM is a special type of luminescent polymer deriving from the intramolecular and intermolecular through-space conjugation effect under polymer chain constraint and intramolecular charge-transfer effect (Jing et al., 2019, Sun et al., 2017). Interestingly, the terminal groups of PTPMs are significant to their luminescence. When the terminal group is a fluoro group, *para*-PTPM is AIE with cyan color, whereas that of trifluoromethyl group is AIE with yellow color. Time-dependent density functional theory calculations indicate terminal groups have significant influences on the band gaps and dihedral angles, which further cause the differences on luminescence (Figures S17 and S18). The band gaps of both F-PTPM and CF_3_-PTPM decrease with the increase of chain length, which indicates the through-space conjugation under polymer chain constraint plays important roles in the luminescent properties. Besides, obviously different HOMO, LUMO, and band gaps are observed between F-PTPMs and CF_3_-PTPMs with different chain lengths, causing the differences in luminescent properties of PTPMs with different terminal groups. SCKP also exhibits polymerization-induced emission property, which can be tuned from ACQ type to AIE type by simply adjusting monomer structures (Figures 4B–4D). The luminescence color of AIE-type polymerization-induced emission can be tuned as well from cyan to yellow by simply adjusting monomer structures and polymerization time. This tunable polymerization-induced emission is due to the formation of luminescent non-conjugated PTPM during polymerization. In comparison, luminescent polymers are generally designed by polymerization of luminescent monomers, where polymerization has a limited effect on the design of luminescent polymers. This result therefore opens up a new avenue to design luminescent polymers through polymerization of nonemissive monomers *in situ*.

### Conclusion

In conclusion, the successful introduction of polarity reversal ketyl strategy into polymer chemistry has been realized through SCKP. In this polarity reversal approach, it exhibits intriguing reversed polymerizability, where traditional excellent leaving groups are not suitable for polymerization but challenging polymerizations involving the cleavage of C-F and C-CF_3_ bonds are realized under mild Barbier conditions. The SCKP involves continuous relay of reactions in the order of carbonyl addition via SET in the formation of ketyl intermediate, C-F activation via ER, and ketyl coupling and C-F cleavage via ER in one polymerization cycle. This SCKP also exhibits intriguing polymerization-induced emission capability to construct luminescent polymers from nonemissive monomers and the emissive properties vary from traditional luminescence to AIE-type luminescence with emission color varying from blue to cyan and yellow, which can be simply adjusted by adjusting the monomer structure and polymerization time. This work therefore provides polymer chemistry with polarity reversal ketyl strategy and opens up a new avenue toward the polymerization of challenging C-X bonds, which might inspire new reaction, polymerization, and luminescent polymer design.

### Limitations of the Study

This study focuses on the demonstration of the introduction of polarity reversal strategy into polymer chemistry to demonstrate the advantage of resplendent polarity reversal mechanism with reversed polymerizability. The substrates used in this study limit on carbonyl compounds, and further investigations on other groups will expand the utilization of this polymerization methodology. Fortunately, carbonyl compounds account for a high portion in organic chemicals and have played significant cornerstone roles in organic chemistry, which might expand the monomer library of polymer materials and inspire the design of novel polymer from carbonyl chemicals.

## Methods

All methods can be found in the accompanying Transparent Methods supplemental file.

## Supplemental Information

Document S1. Transparent Methods, Figures S1–S32, and Tables S1–S4
