# Supplementary material for: Barbier Self-Condensing Ketyl Polymerization-Induced Emission: A Polarity Reversal Approach to Reversed Polymerizability
Source: iScience. 2020 Apr 5;23(4):101031. doi: 10.1016/j.isci.2020.101031 (PMC7160573; doi:10.1016/j.isci.2020.101031)
Supplement: Document S1. Transparent Methods, Figures S1–S32, and Tables S1–S4 [file mmc1.pdf]

iScience, Volume *23*

Supplemental Information

Barbier Self-Condensing Ketyl Polymerization-Induced Emission: A Polarity Reversal Approach to Reversed Polymerizability

Shun-Shun Li, Nengbo Zhu, Ya-Nan Jing, Yajun Li, Hongli Bao, and Wen-Ming Wan

Supplemental Figures

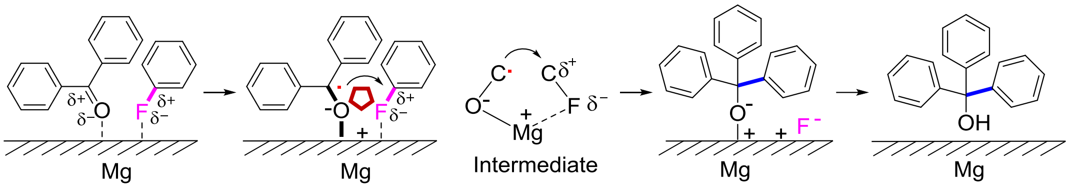

Figure S1. Reductive polarity reversal ketyl mechanism of Mg-mediated C-F bond activation-cleavage, related to **Figure 1.**

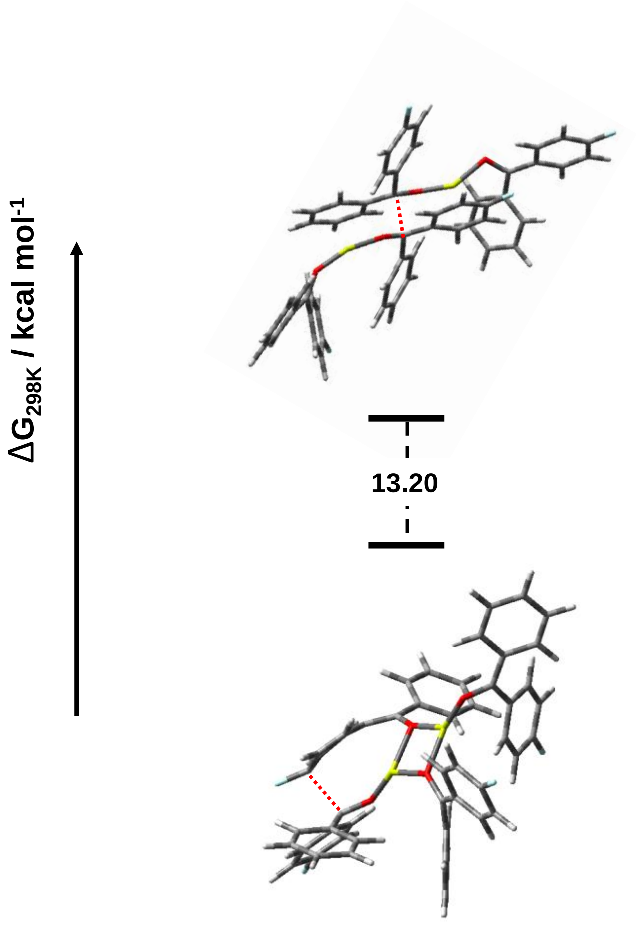

Figure S2. Gibbs free energy profile for the pinacol coupling reaction pathway and radical coupling reaction pathway via electron resonance. Relative free energies are in kcal/mol, related to **Figure 1.**

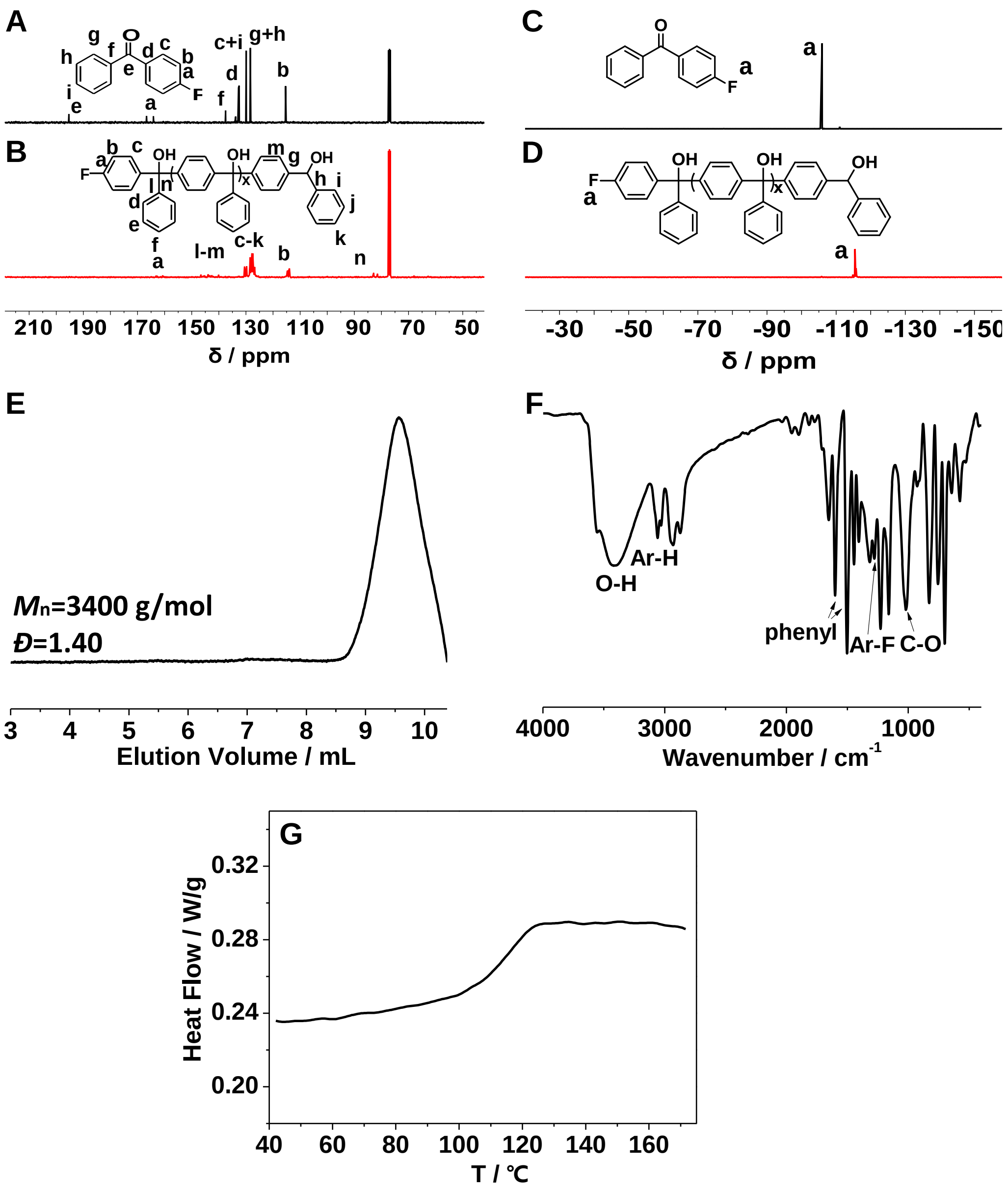

Figure S3. ^{13}C NMR spectra of 4-fluorobenzophenone (A) and fluoro-PTPM (B) in $CDCl_3$, ^{19}F NMR spectra of 4-fluorobenzophenone (C) and fluoro-PTPM (D) in $CDCl_3$, GPC curve (E), FT-IR spectrum (F) and DSC curve (G) of fluoro-PTPM (Tg ~104.7 ^{o}C), related to **Figure 2.**

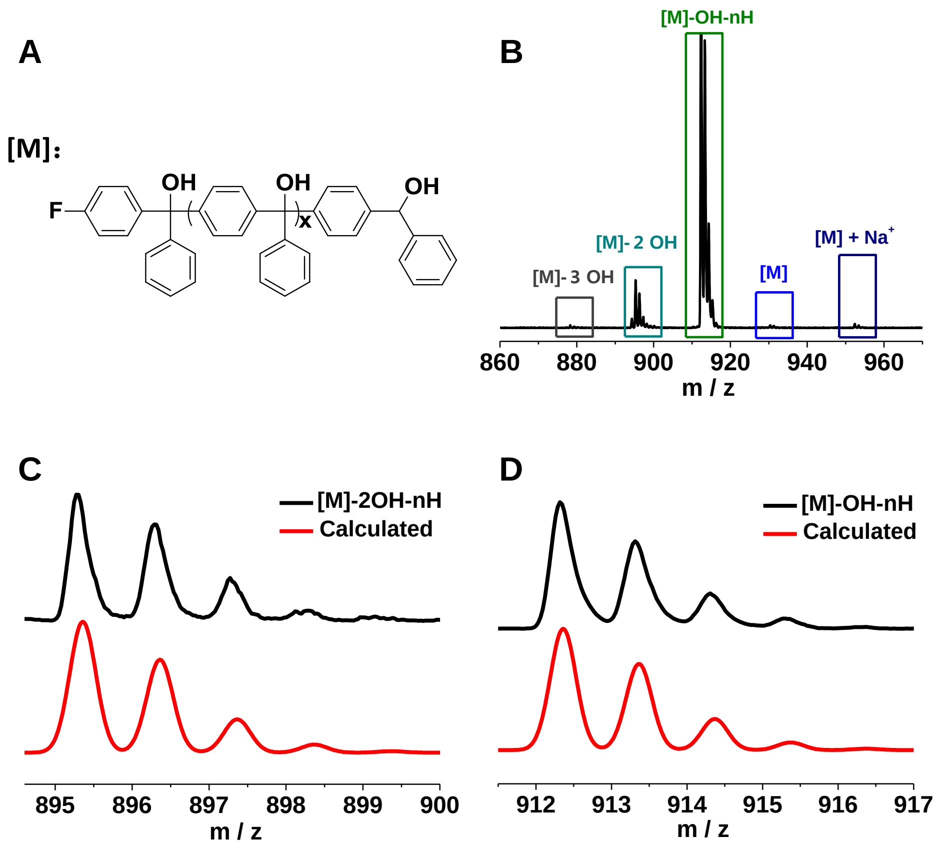

Figure S4. MALDI-TOF characterization of fluoro-PTPM. Chemical structure of fluoro-PTPM (A), enlarged MALDI-TOF spectrum of pentamer (B), comparison of [M]-2OH-nH (DP=5) between observed and calculated spectra (C), comparison of [M]-OH-nH (DP=5) between observed and calculated spectra (D), related to **Figure 2.**

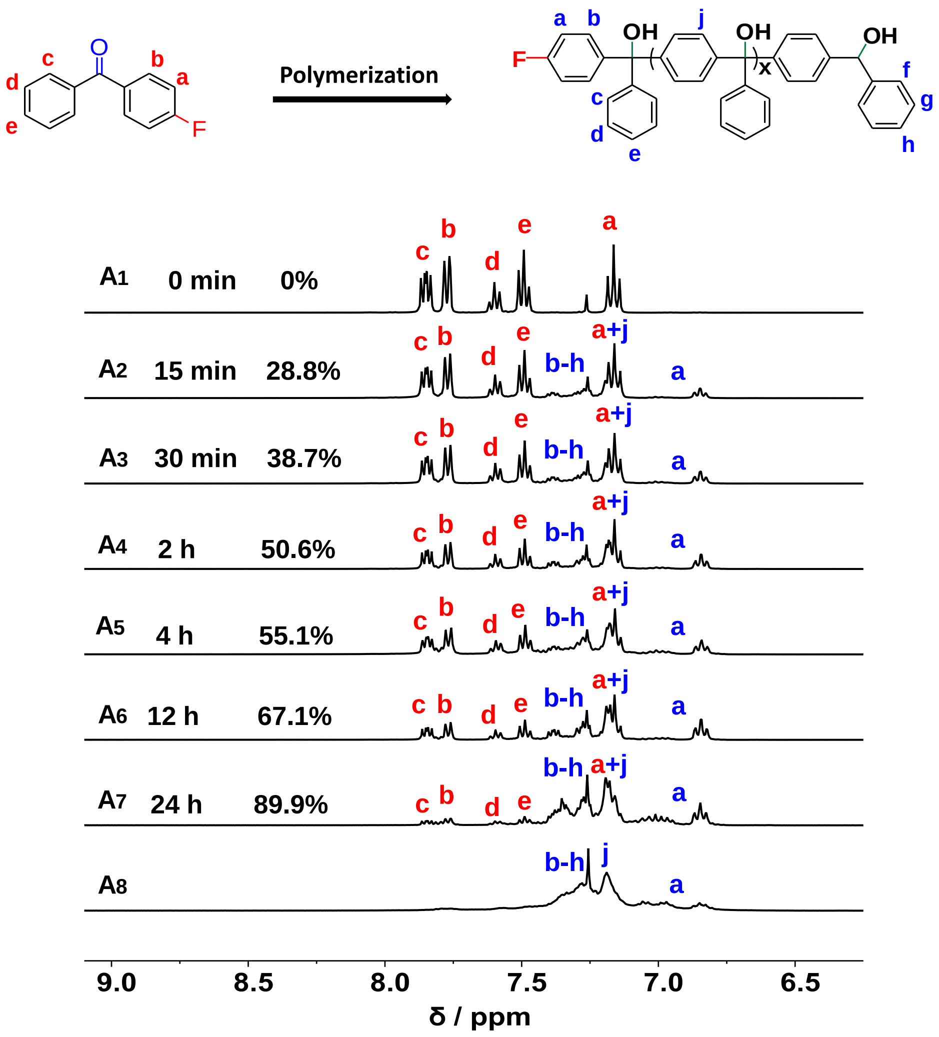

Figure S5. Polymerization process traced by ^{1}H NMR spectra: monomer (A1), 15min (A2), 30min (A3), 2h (A4), 4h (A5), 12h (A6), 24h (A7) and after precipitation (A8), related to **Figure 2.**

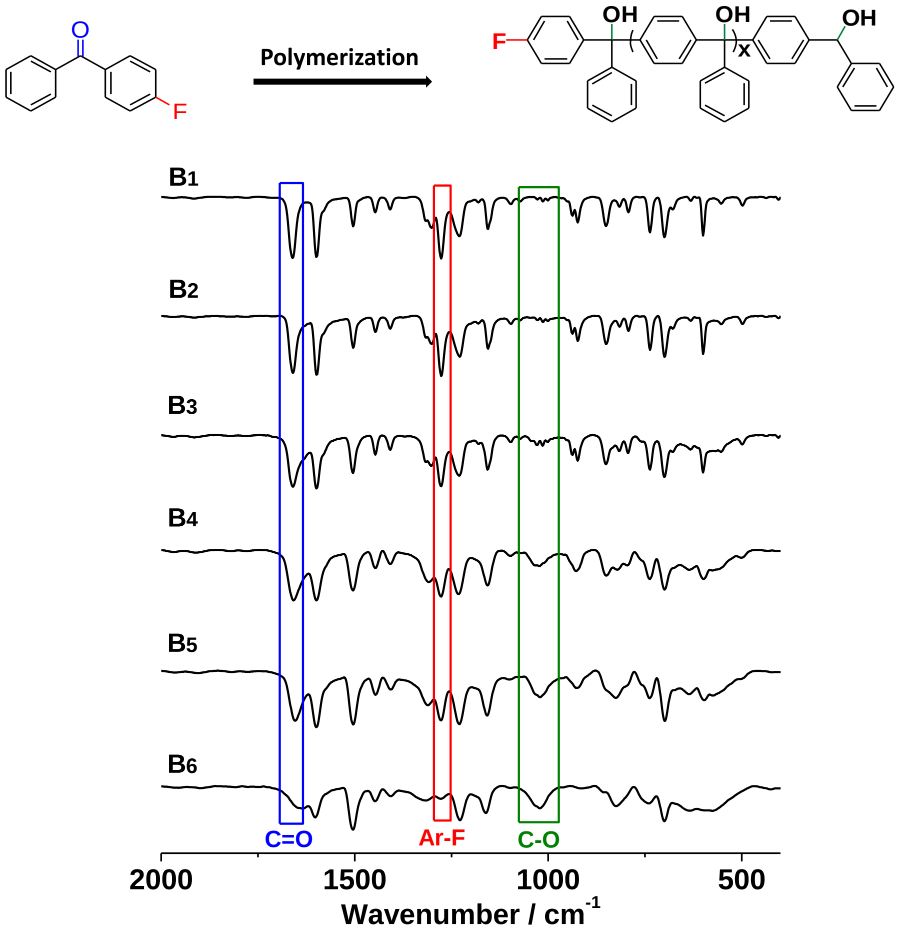

Figure S6. Polymerization process traced by FT-IR spectra: 15min (B1), 30min (B2), 2h (B3), 4h (B4), 12h (B5), 24h (B6), related to **Figure 2.**

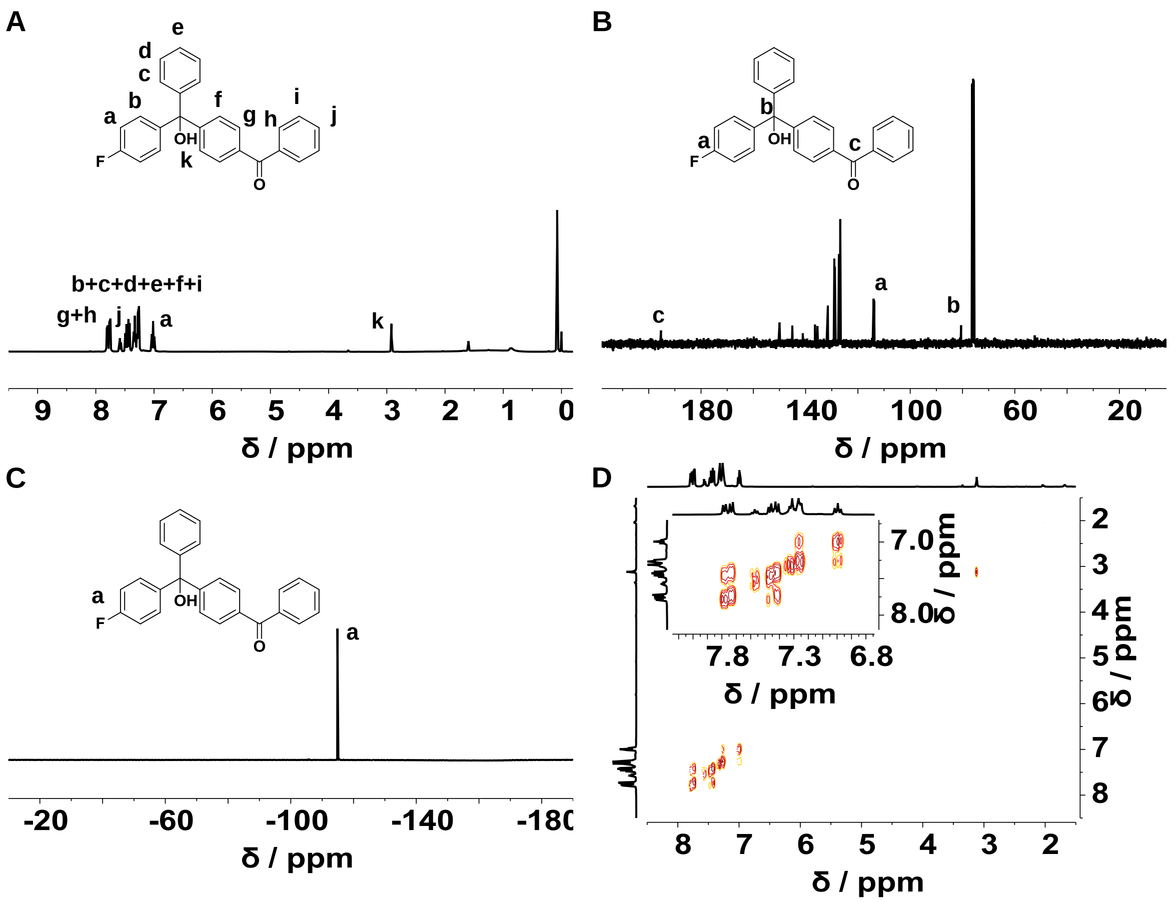

Figure S7. Structural characterizations of isolated dimer intermediate: ^{1}H NMR spectrum (A), ^{13}C NMR spectrum (B), ^{19}F NMR spectrum and ^{1}H-^{1}H COSY NMR spectrum (D), related to **Figure 4.**

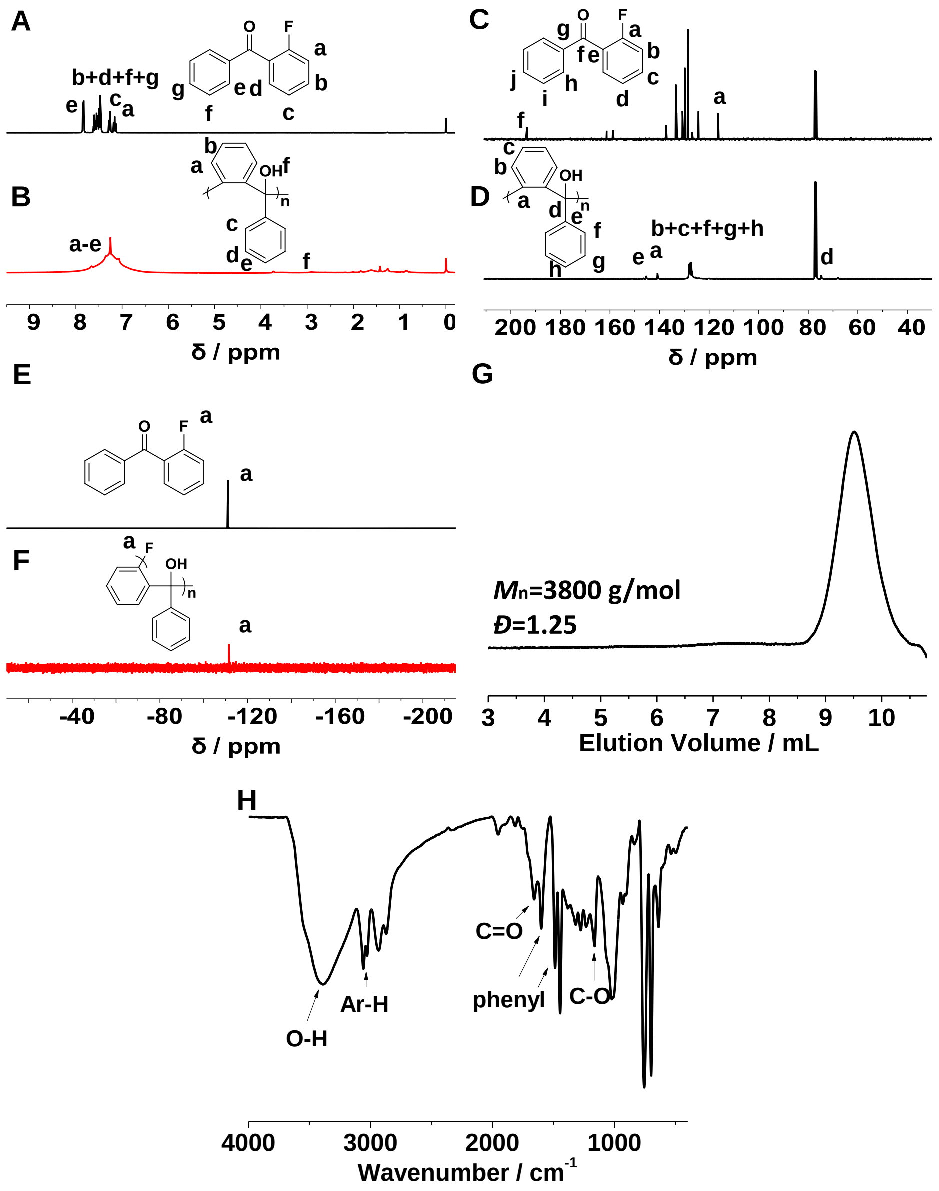

Figure S8. Structural characterization of 2-fluoro-PTPM: ^{1}H NMR spectra of 2-fluorobenzophenone (A) and 2-fluoro-PTPM (B) in $CDCl_3$, ^{13}C NMR spectra of 2-fluorobenzophenone (C) and 2-fluoro-PTPM (D) in $CDCl_3$, ^{19}F NMR spectra of 2-fluorobenzophenone (E) and 2-fluoro-PTPM (F) in $CDCl_3$,GPC curve (G) and FT-IR spectrum (H) of 2-fluoro-PTPM, related to **Figure 4.**

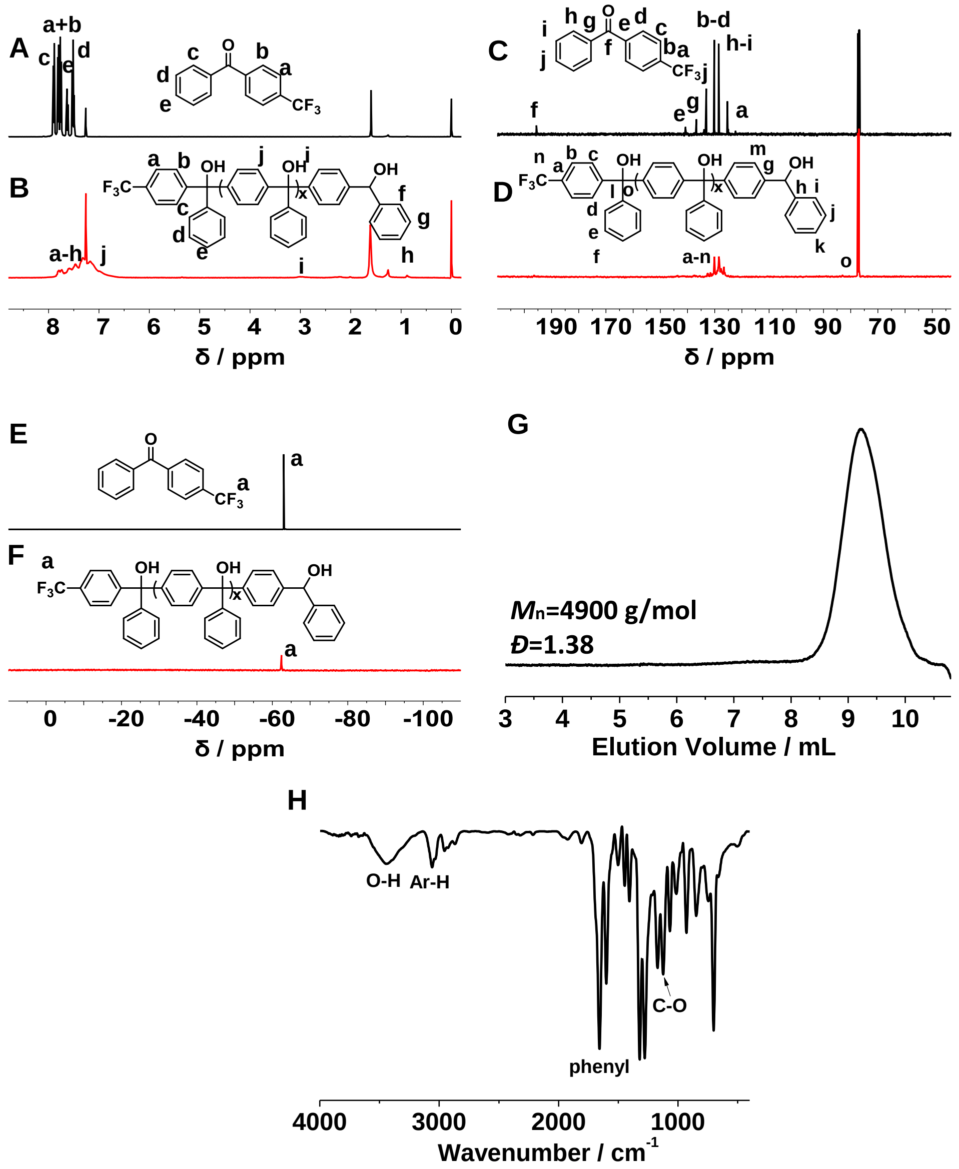

Figure S9. Structural characterization of CF_3-PTPM: ^{1}H NMR spectra of 4-trifluoromethylbenzophenone (A) and CF_3-PTPM (B) in $CDCl_3$, ^{13}C NMR spectra of 4-trifluoromethylbenzophenone (C) and CF_3-PTPM (D) in $CDCl_3$, ^{19}F NMR spectra of 4-trifluoromethylbenzophenone (E) and CF_3-PTPM (F) in $CDCl_3$, GPC curve (G) and FT-IR spectrum (H) of CF_3-PTPM, related to **Figure 4.**

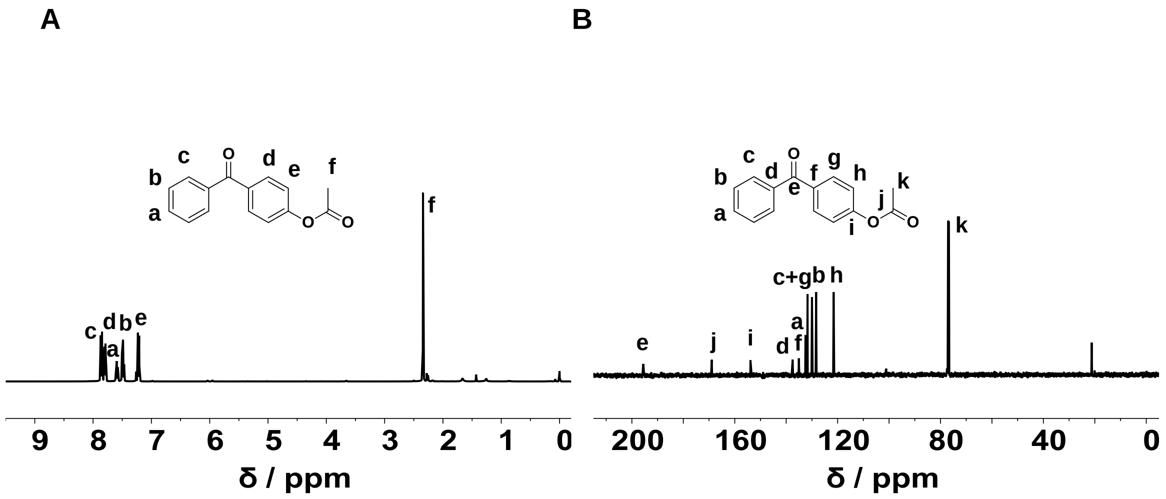

Figure S10. Structural characterization of 4-acetoxybenzophenone: ^{1}H NMR spectrum (A) and ^{13}C NMR spectrum (B), related to **Figure 4.**

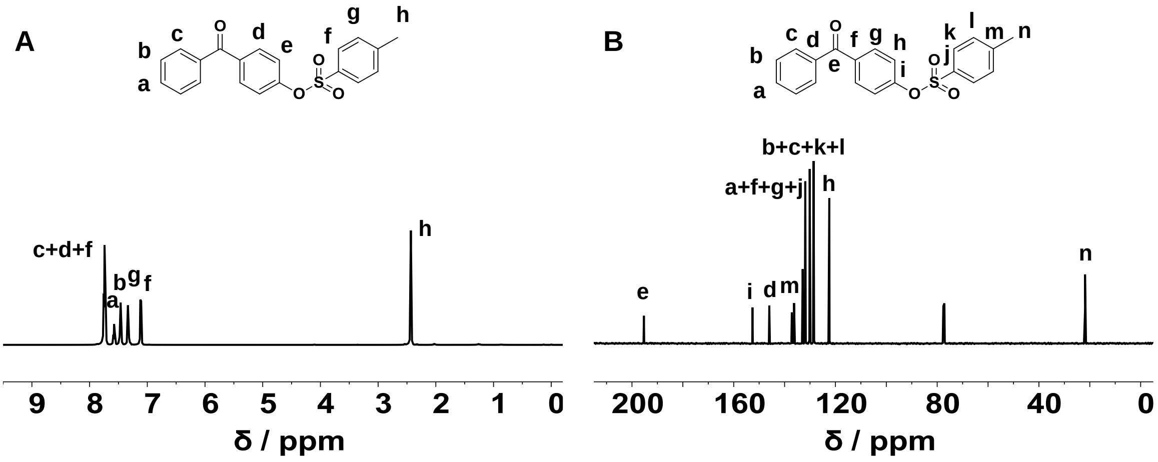

Figure S11. Structural characterization of 4-toluenesulfonylbenzophenone: ^{1}H NMR spectrum (A) and ^{13}C NMR spectrum (B), related to **Figure 4.**

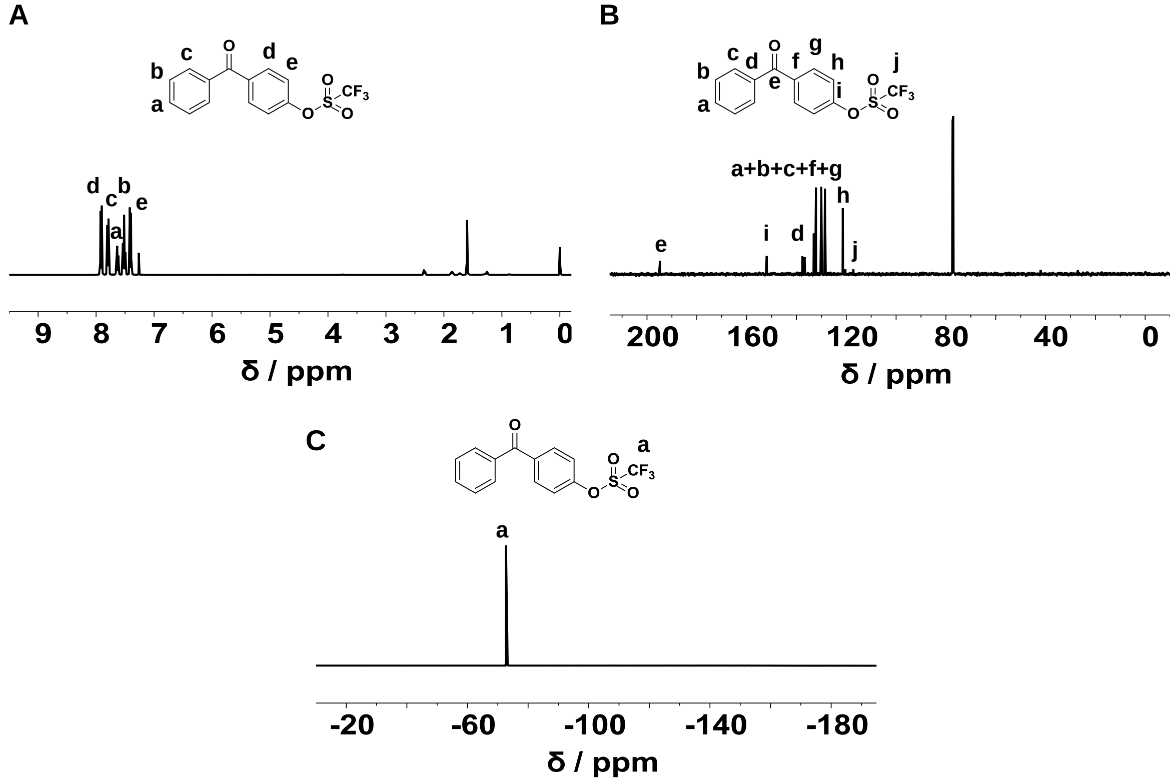

Figure S12. Structural characterization of 4-trifluoromathanesulfonylbenzophenone: ^{1}H NMR spectrum (A), ^{13}C NMR spectrum (B) and ^{19}F NMR spectrum (C), related to **Figure 4.**

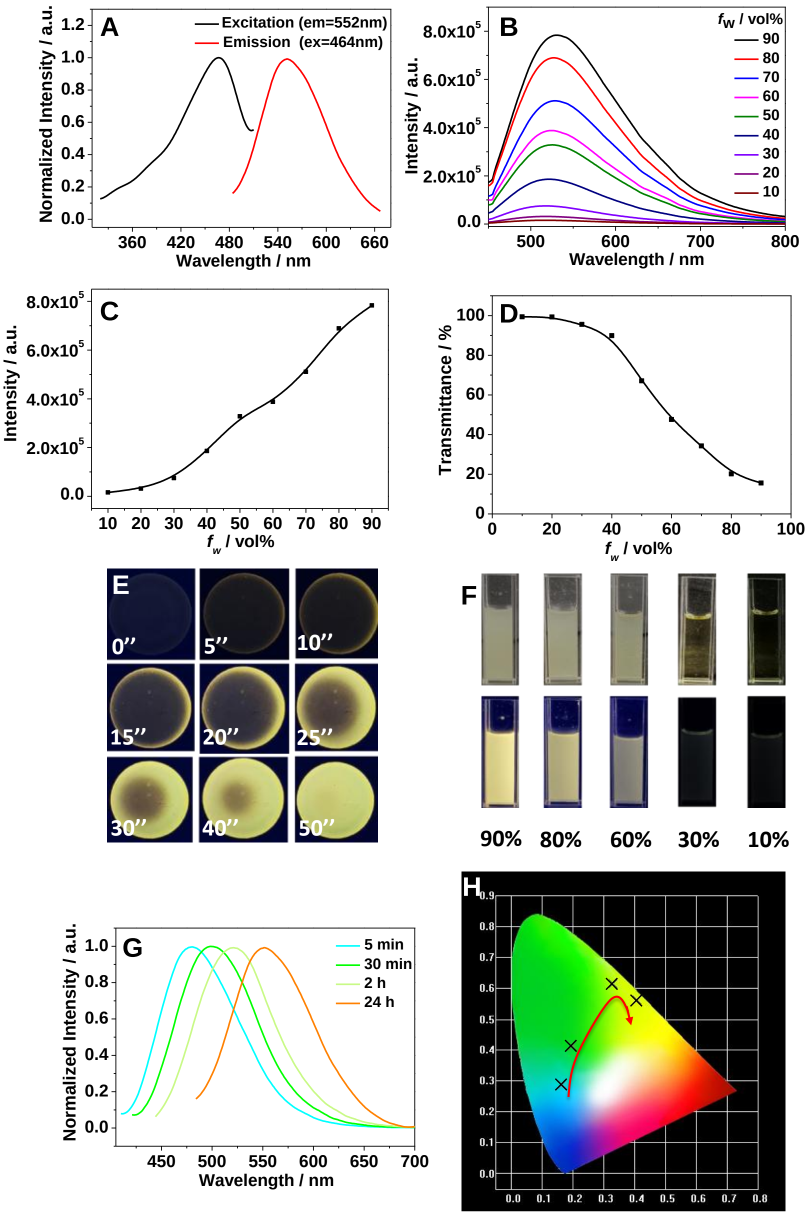

Figure S13. Fluorescence properties of PTPMs. Excitation and emission spectra of CF_3-PTPM in the solid state (A). Emission spectra of CF_3-PTPM (0.1 mg/mL) in water/THF mixtures with different water volume fractions (*fw*, vol %) (excited at 425 nm) (B). Plots of emission intensities of CF_3-PTPM (0.1 mg/mL) in water/THF mixtures with different water volume fractions (*fw*, vol %) (excited at 425 nm) (C). Transmittance of CF_3-PTPM (0.1 mg/mL) in water/THF mixtures with different water volume fractions (*fw*, vol %) at 650 nm wavelength (D). Digital photos of one drop of CF_3-PTPM solution (10 mg/mL in THF) on thin-layer chromatography at different times (time unit is second) of evaporation (under UV irradiation at 365 nm) (E). Digital photos of CF_3-PTPM (0.1 mg/mL) in water/THF mixtures with different water volume fractions (*fw*, vol %) (under sunlight and UV irradiation at 365 nm) (F). Emission spectra of CF_3-PTPM obtained at different polymerization times (G). CIE coordinates of CF_3-PTPM obtained at different polymerization times (H), related to **Figure 4.**

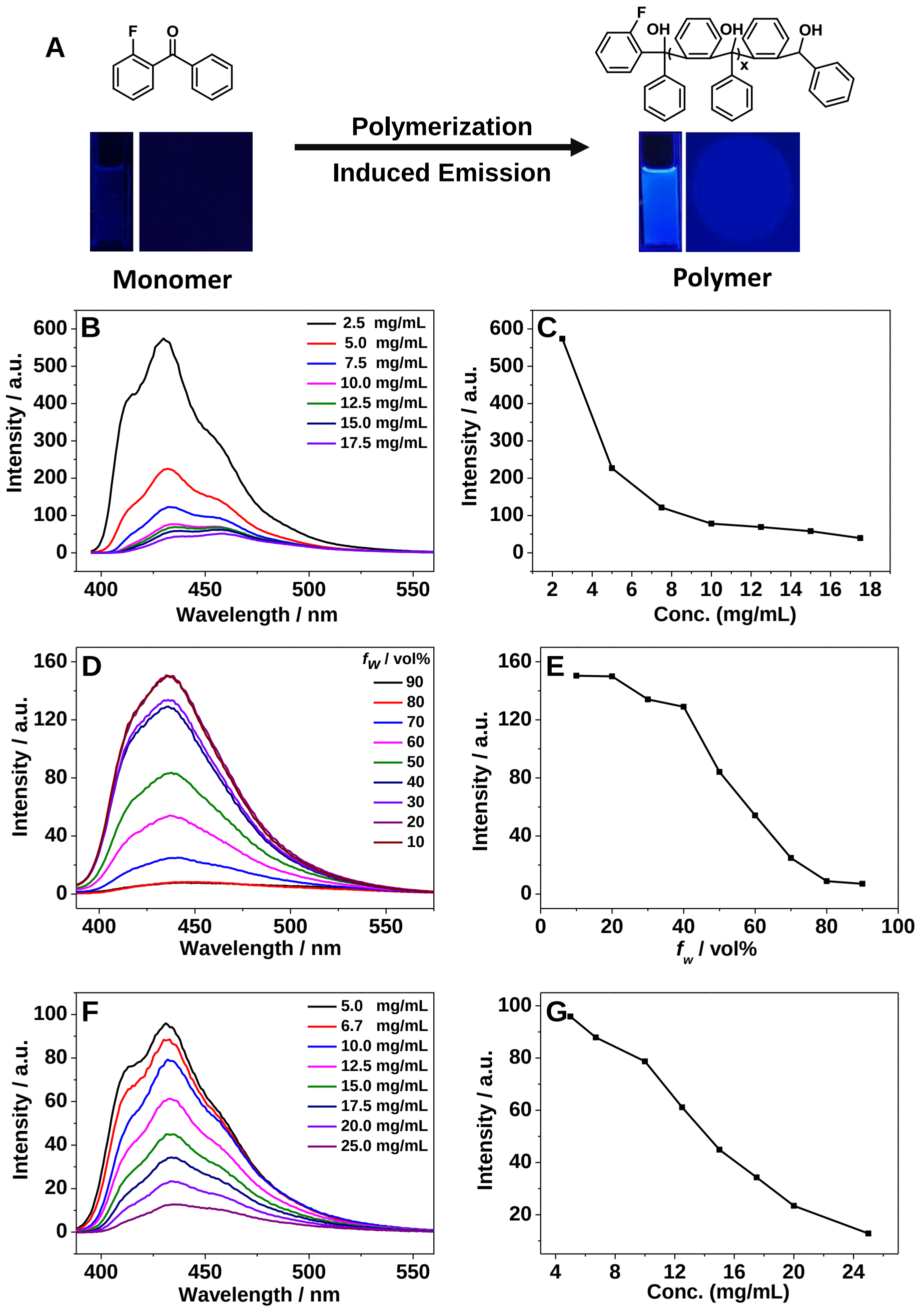

Figure S14. Photophysical properties of 2-fluoro-PTPM solution in THF. Chemical structure and emission digital photos (under irradiation with UV lamp at 365 nm) of polymerization process of 2-fluorobenzophenone (A), emission spectra at different concentration (excited at 375 nm) (B) and plots of fluorescent intensity (C) of polymers at a polymerization time of 12 h, emission spectra of 2-fluoro-PTPM (0.1 mg/mL) in water/THF mixtures with different water volume fractions (f_w, vol%)) (D) and plots of fluorescent intensities (E) of polymers at a polymerization time of 24 h, emission spectra at different concentration (excited at 375 nm) (F) and plots of fluorescent intensity (G) of polymers at a polymerization time of 24 h, related to **Figure 4.**

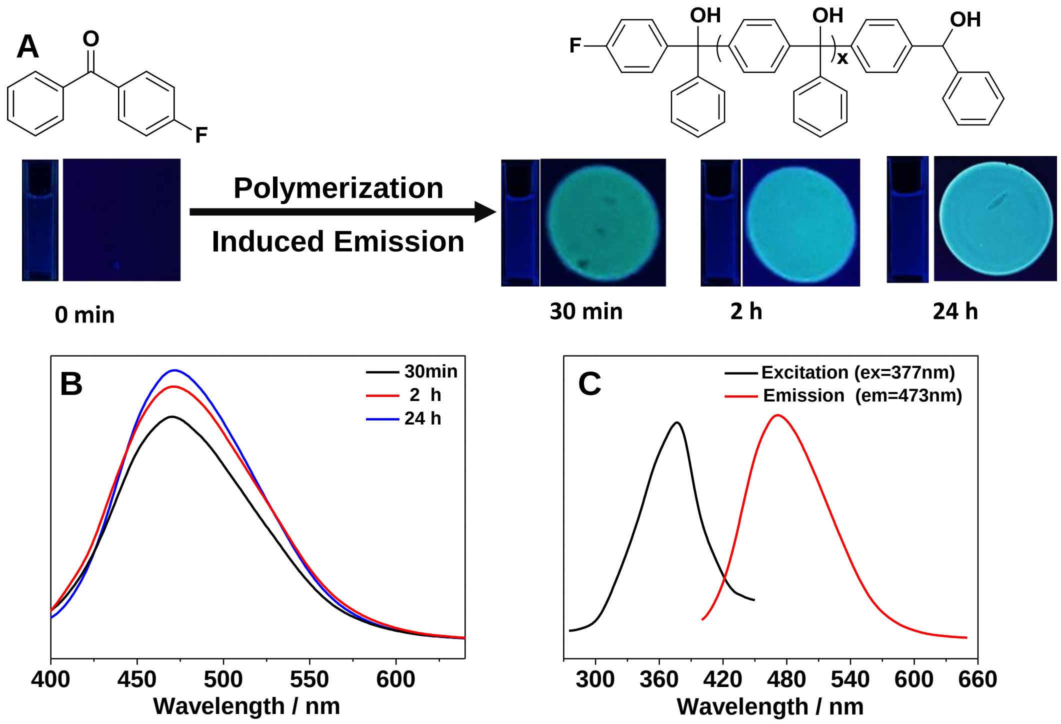

Figure S15. Polymerization-induced emission property of fluoro-PTPM. Emission digital photos (under irradiation with UV lamp at 365 nm) of polymerization process of 4-fluorobenzophenone at different polymerization times (A), emission spectra of fluoro-PTPM obtained at different polymerization times and digital photos (under UV irradiation at 365 nm) (B), excitation and emission spectra in the solid state (C), related to **Figure 4.**

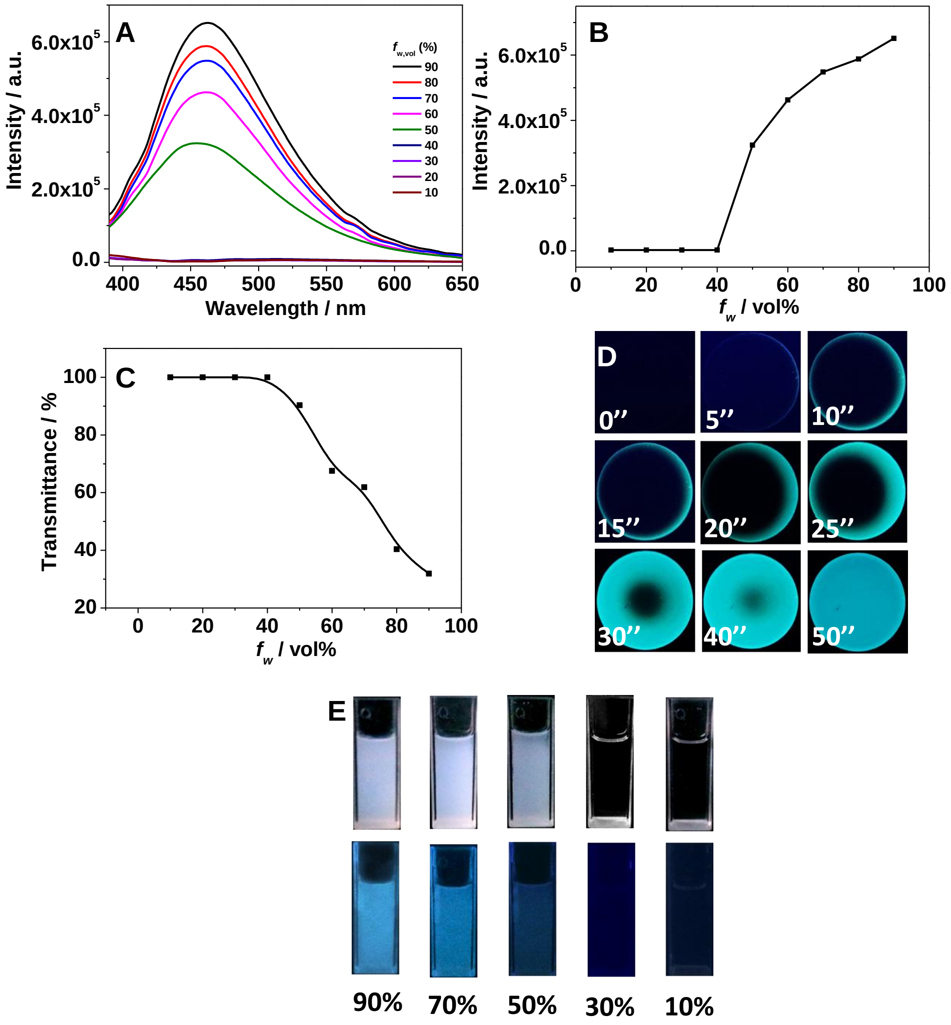

Figure S16. Aggregation-induced emission property of fluoro-PTPM. Emission spectra of fluoro-PTPM (0.1 mg/mL) in water/THF mixtures with different water volume fractions (f_w, vol %) (excited at 440 nm) (A), plots of emission intensities of fluoro-PTPM (0.1 mg/mL) in water/THF mixtures with different water volume fractions (f_w, vol %) (excited at 440 nm) (B), transmittance of fluoro-PTPM (0.1 mg/mL) in water/THF mixtures with different water volume fractions (f_w, vol %) at 650 nm wavelength (C), digital photos of one drop of fluoro-PTPM solution (10 mg/mL in THF) on thin-layer chromatography at different times (time unit is second) of evaporation (under UV irradiation at 365 nm) (D), digital photos of fluoro-PTPM (0.1 mg/mL) in water/THF mixtures with different water volume fractions (f_w, vol %) (under sunlight and UV irradiation at 365 nm) (F), related to **Figure 4.**

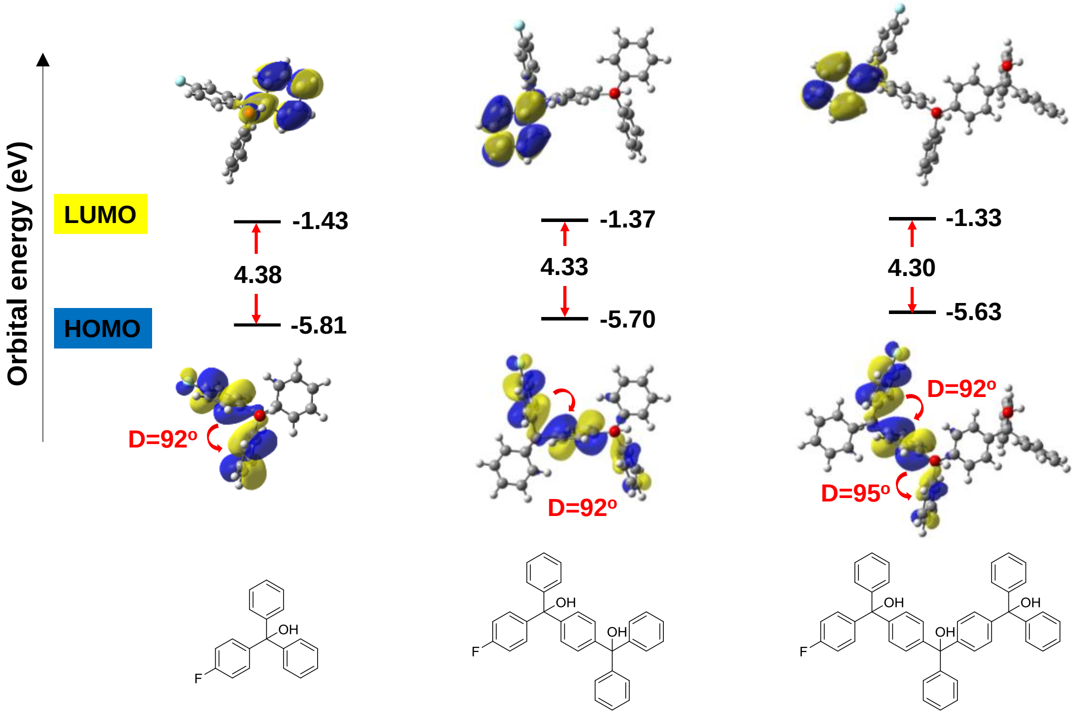

Figure S17. Electron cloud distributions and energy levels (eV) of fluoro-TPM, fluoro-TPM dimer and fluoro-TPM trimer in the excited state calculated by TD-DFT B3LYP/6-311G*, Gaussian 09 program. D: dihedral angle, related to **Figure 4.**

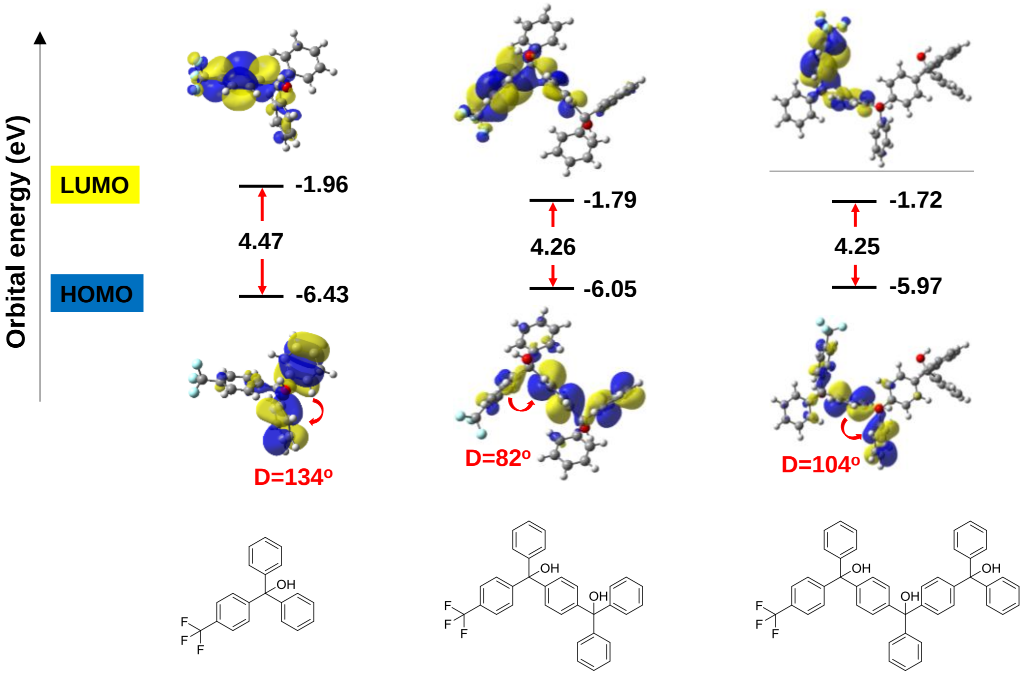

Figure S18. Electron cloud distributions and energy levels (eV) of trifluoromethyl-TPM, trifluoromethyl-TPM dimer and trifluoromethyl-TPM trimer in the excited state calculated by TD-DFT B3LYP/6-311G*, Gaussian 09 program. D: dihedral angle, related to **Figure 4.**

NMR Spectra

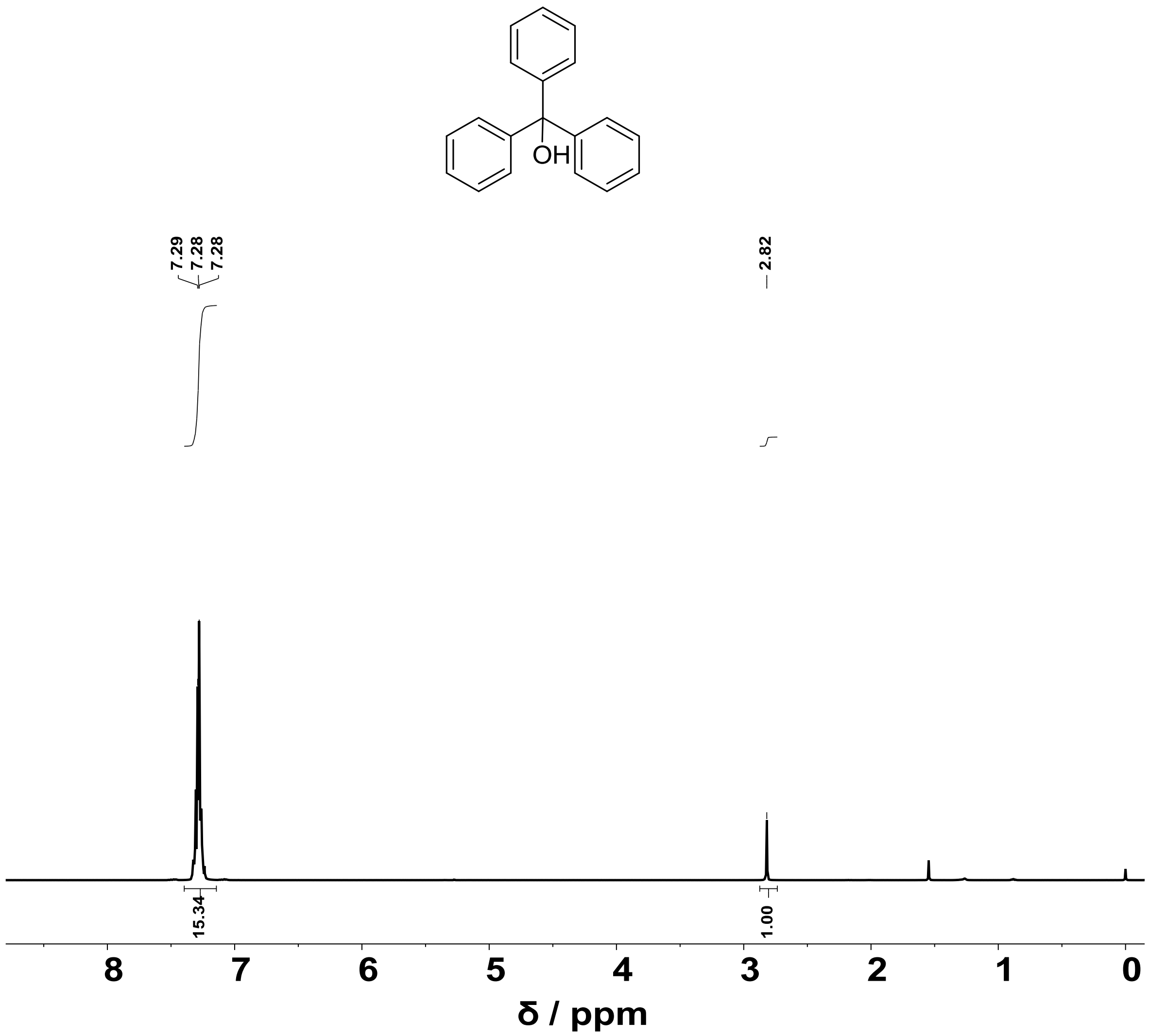

Figure S19. ^{1}H NMR spectrum of compound **TPM**, related to **Figure 1.**

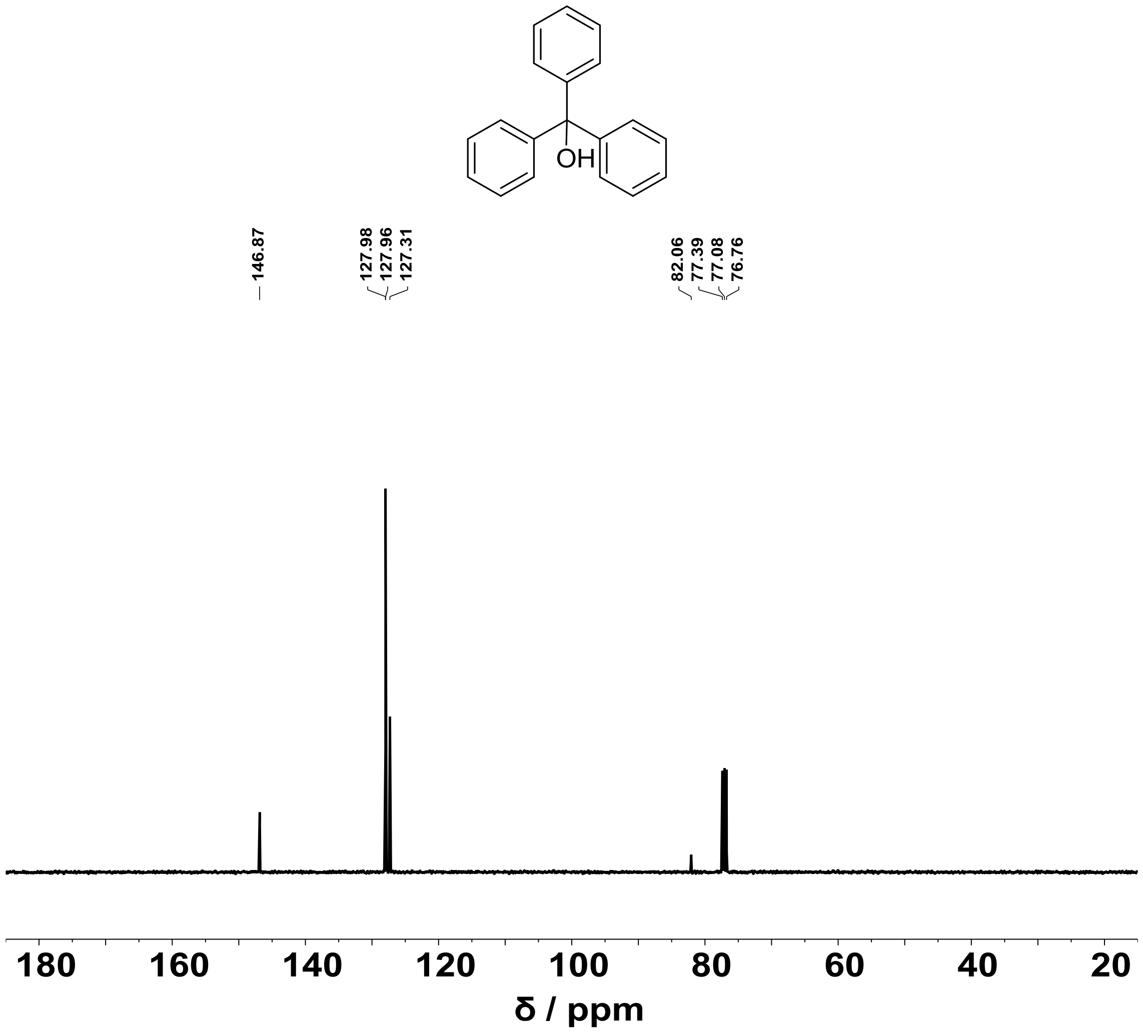

Figure S20. ^{13}C NMR spectrum of compound **TPM**, related to **Figure 1.**

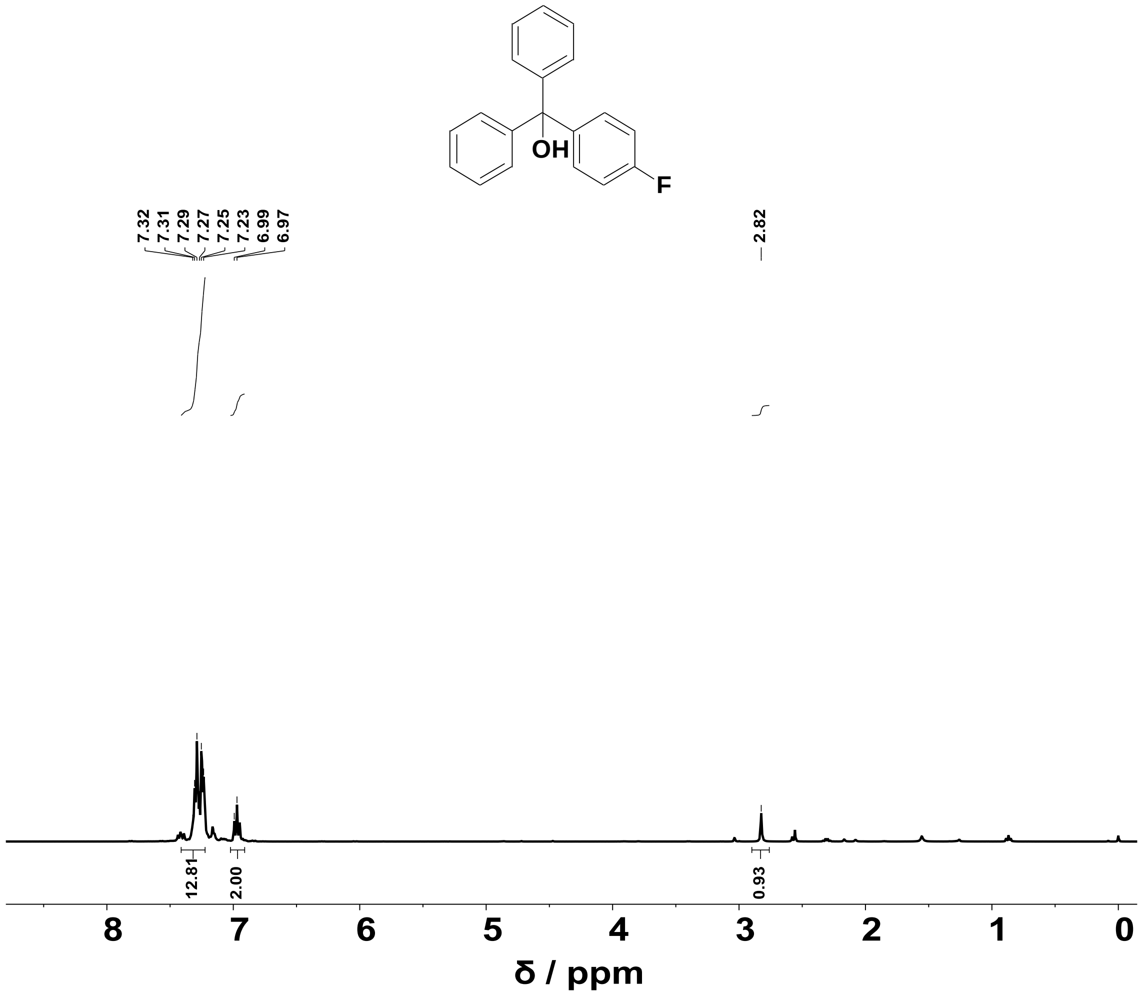

Figure S21. ^{1}H NMR spectrum of compound **F-TPM**, related to **Figure 1.**

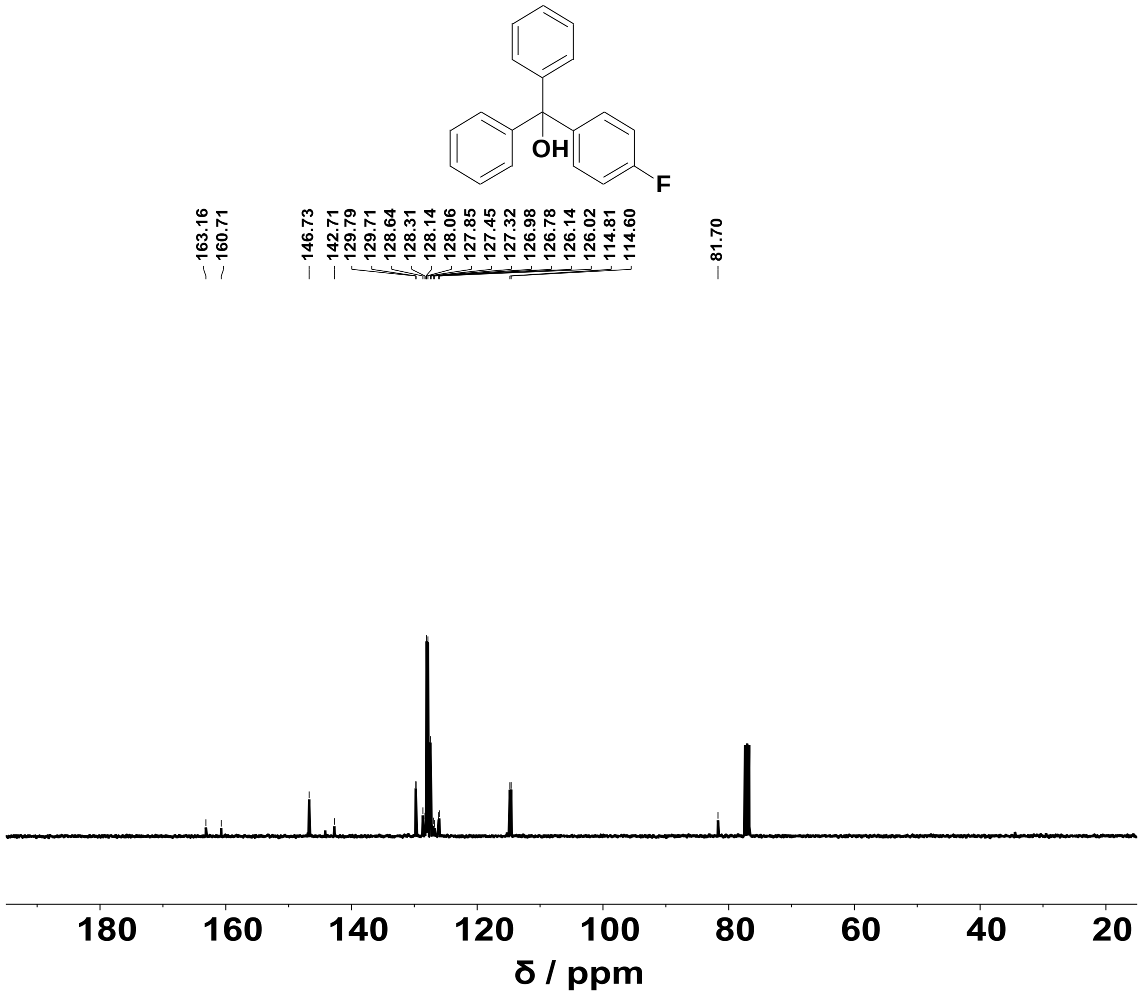

Figure S22. ^{13}C NMR spectrum of compound **F-TPM**, related to **Figure 1.**

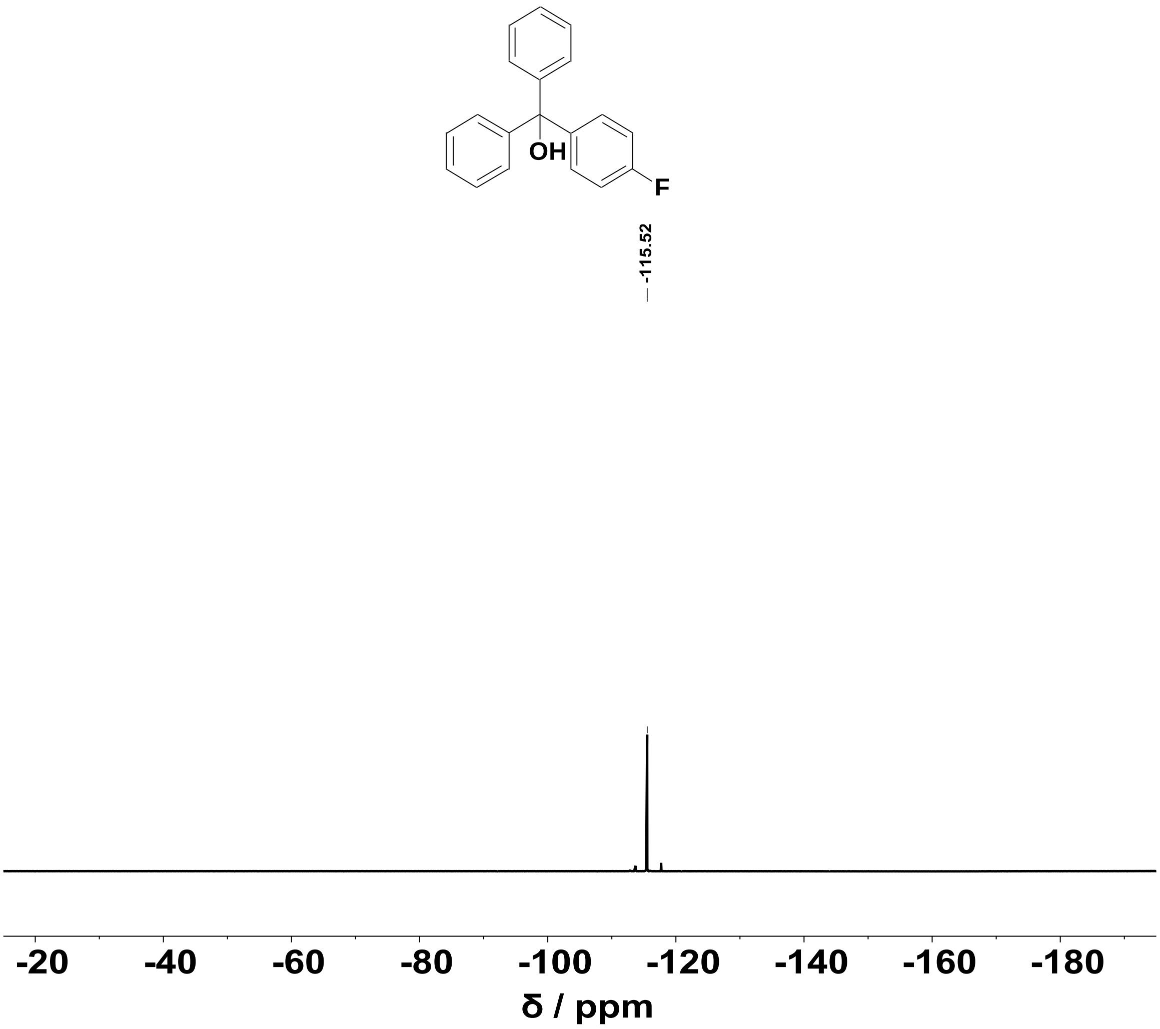

Figure S23. ^{19}F NMR spectrum of compound **F-TPM**, related to **Figure 1.**

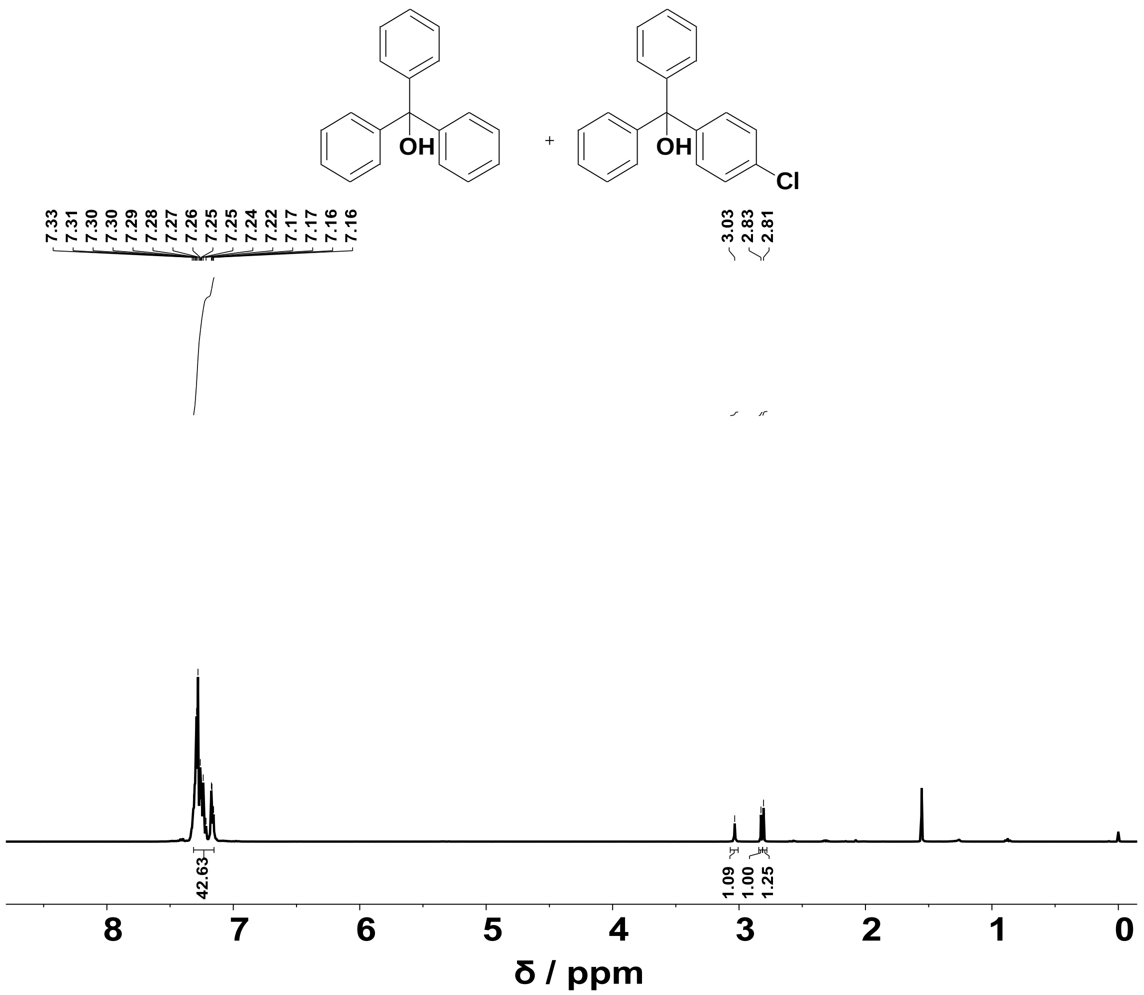

Figure S24. ^{1}H NMR spectrum of compound **TPM** and **Cl-TPM**, related to **Figure 1.**

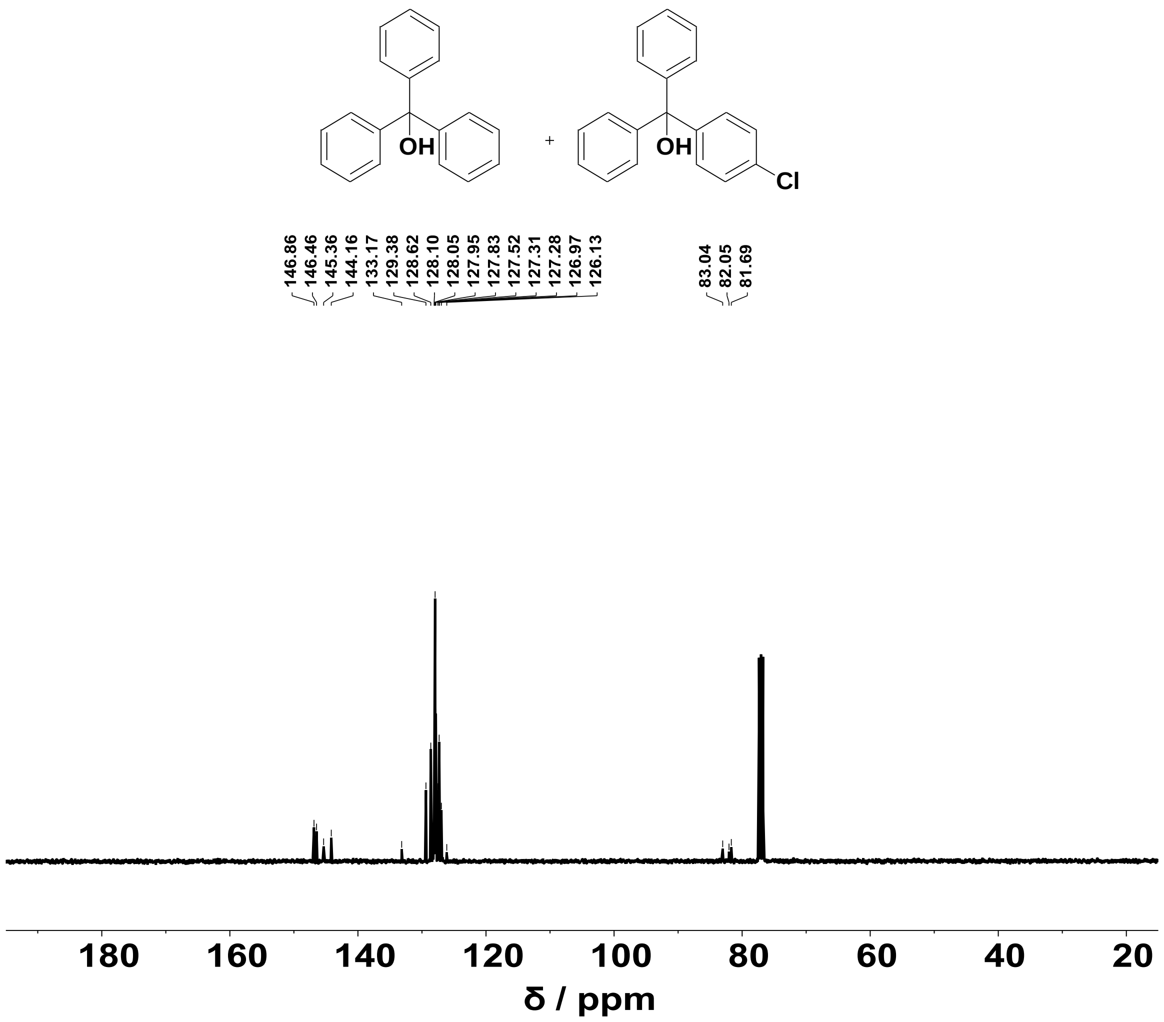

Figure S25. ^{13}C NMR spectrum of compound **TPM** and **Cl-TPM**, related to **Figure 1.**

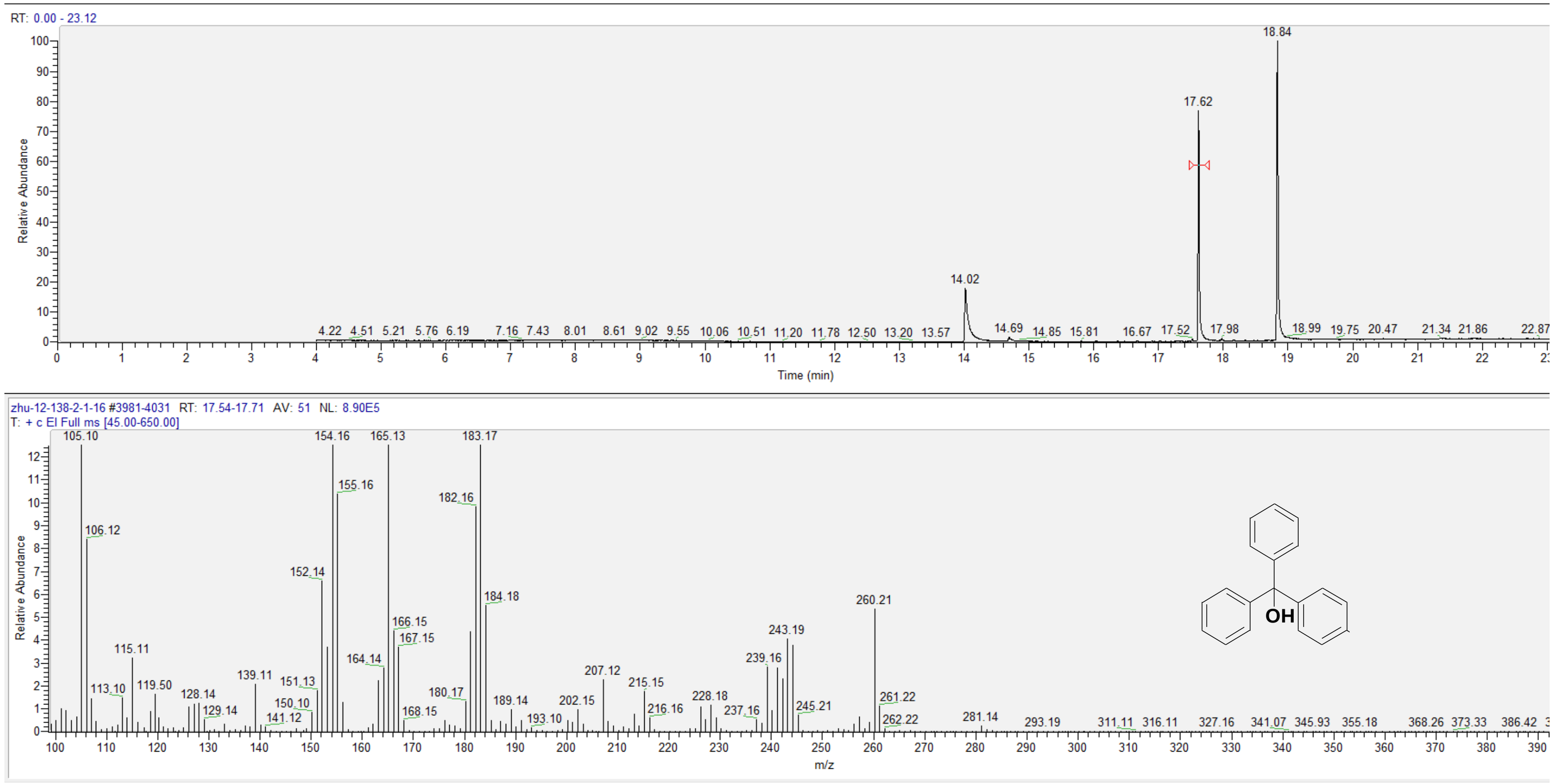

Figure S26. GC-MS characterization of compound **TPM**, related to **Figure 1.**

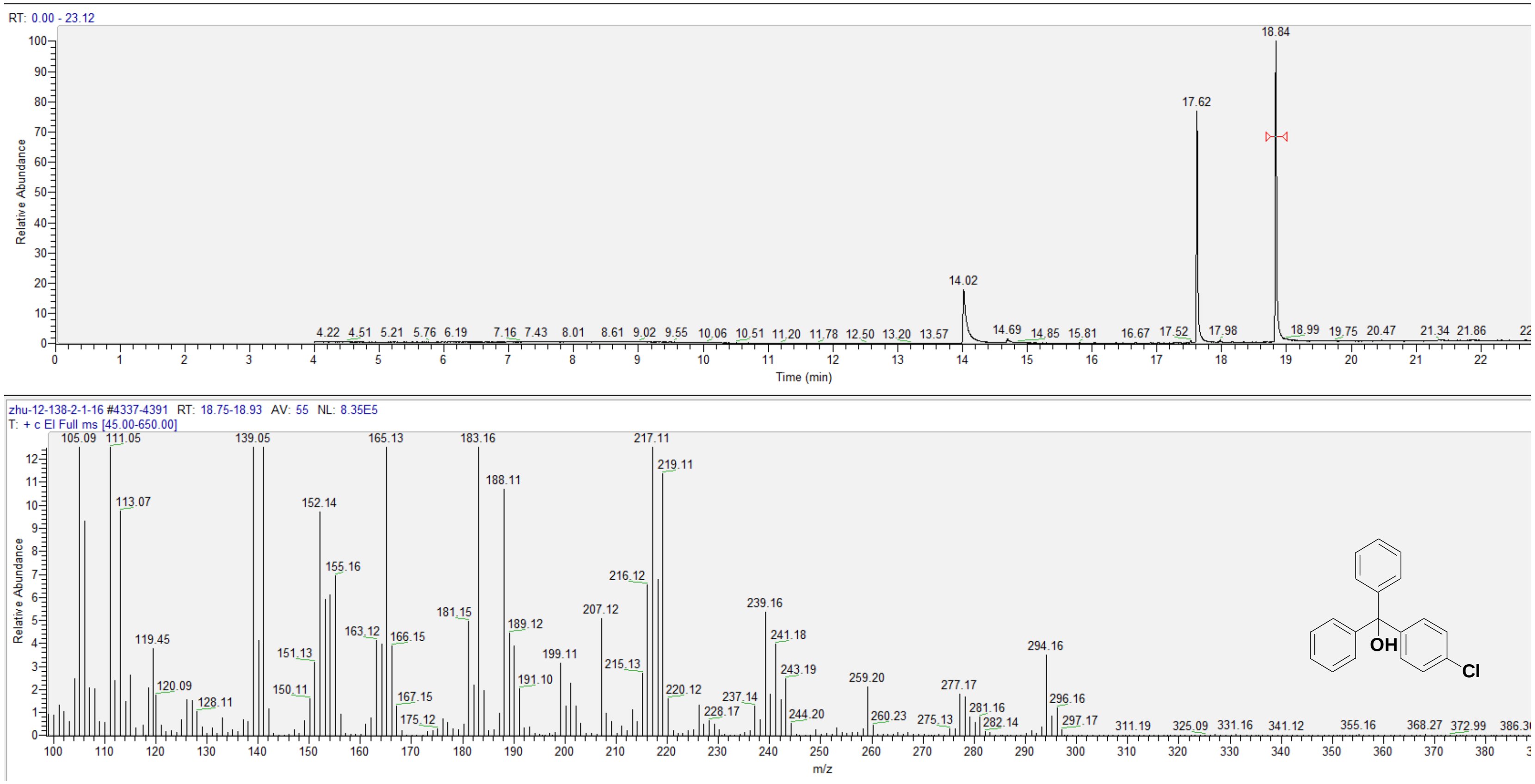

Figure S27. GC-MS characterization of compound **Cl-TPM**, related to **Figure 1.**

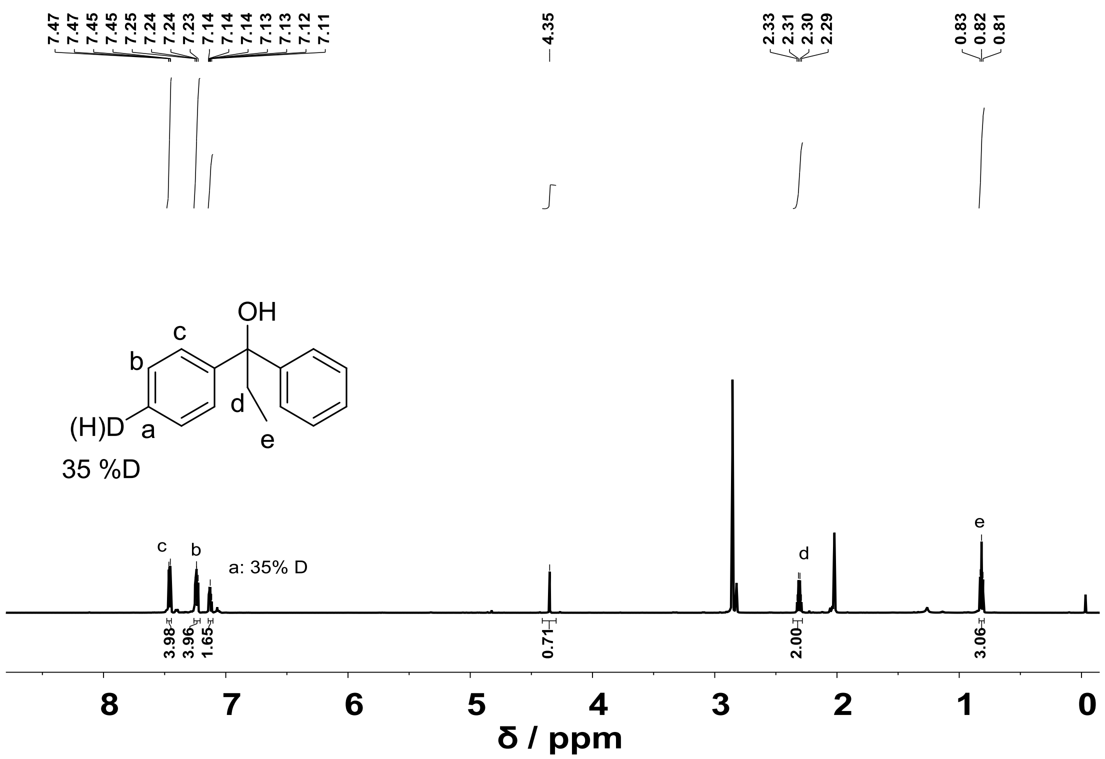

Figure S28. ^{1}H NMR spectrum of *para*-deuterated diphenylethylmethanol, related to **Figure 3.**

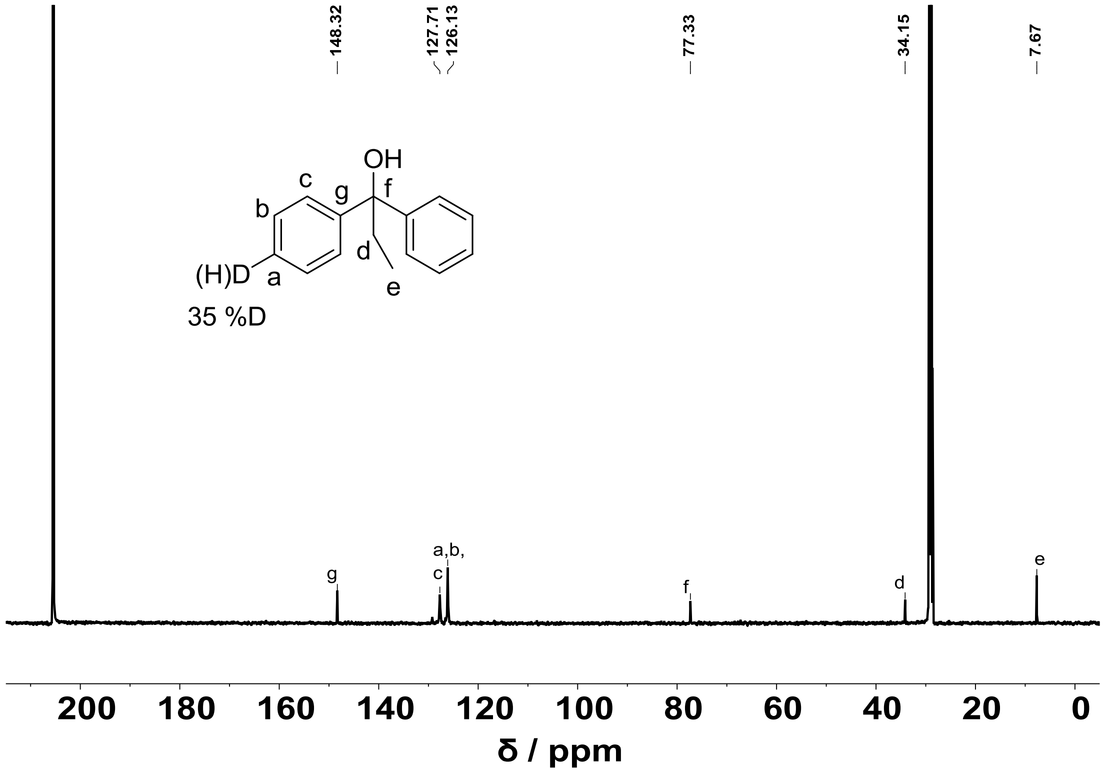

Figure S29. ^{13}C NMR spectrum of *para*-deuterated diphenylethylmethanol, related to **Figure 3.**

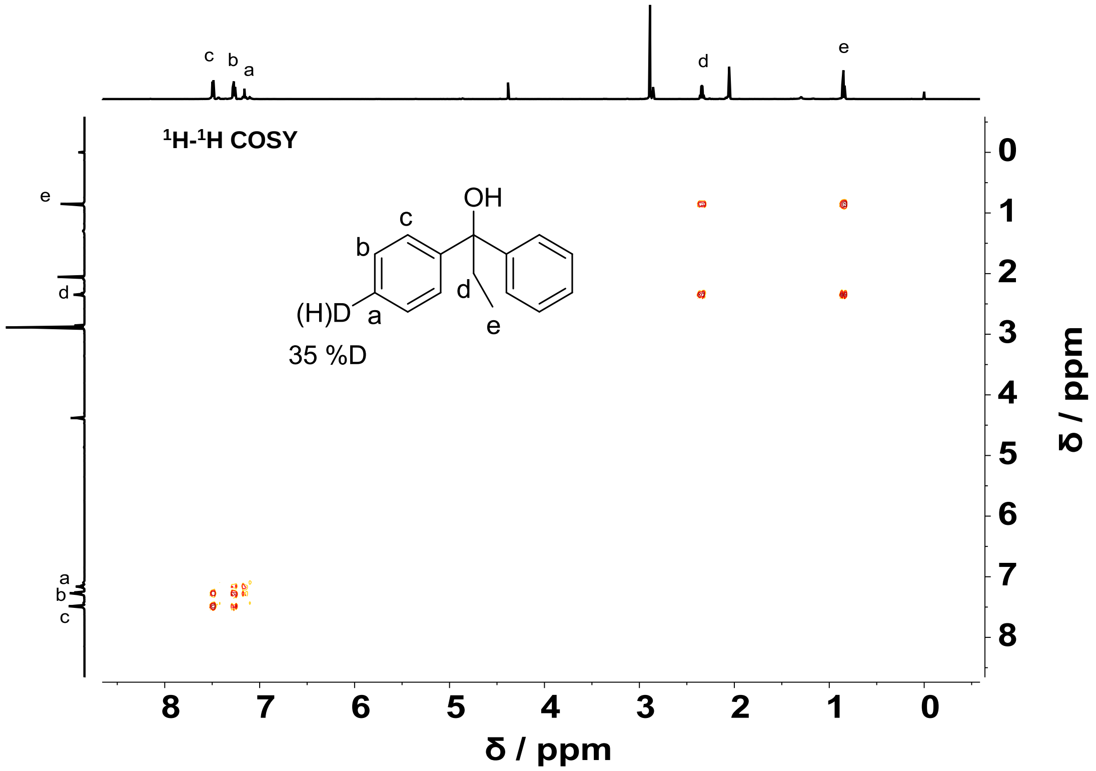

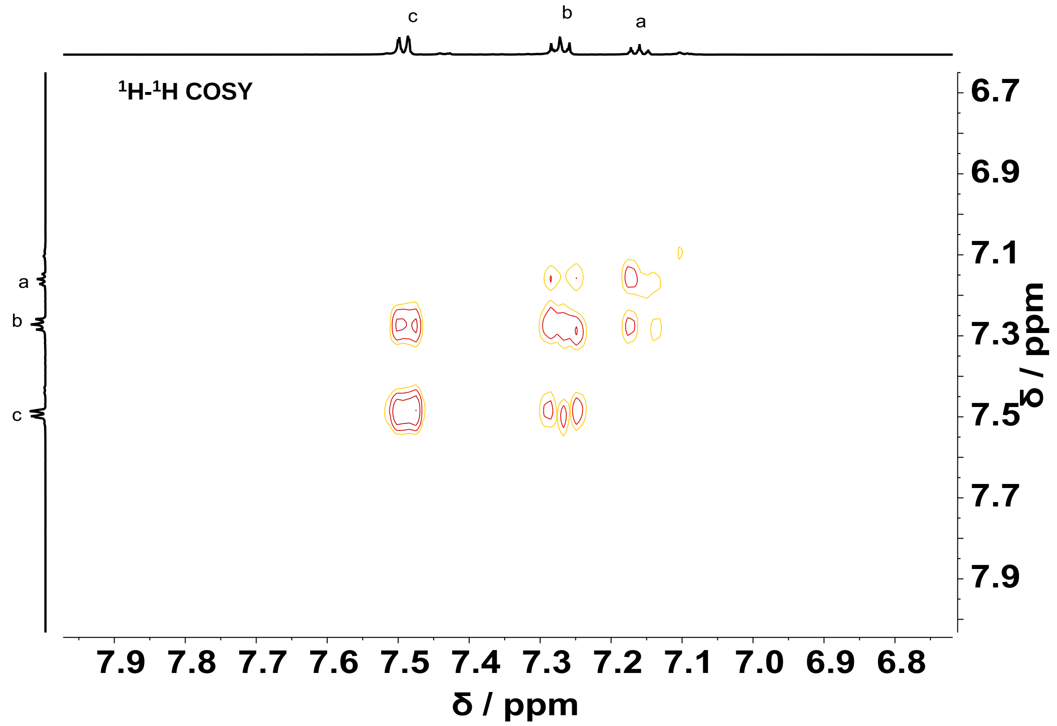

Figure S30. ^{1}H-^{1}H COSY NMR spectra of para-deuterated diphenylethylmethanol, related to **Figure 3.**

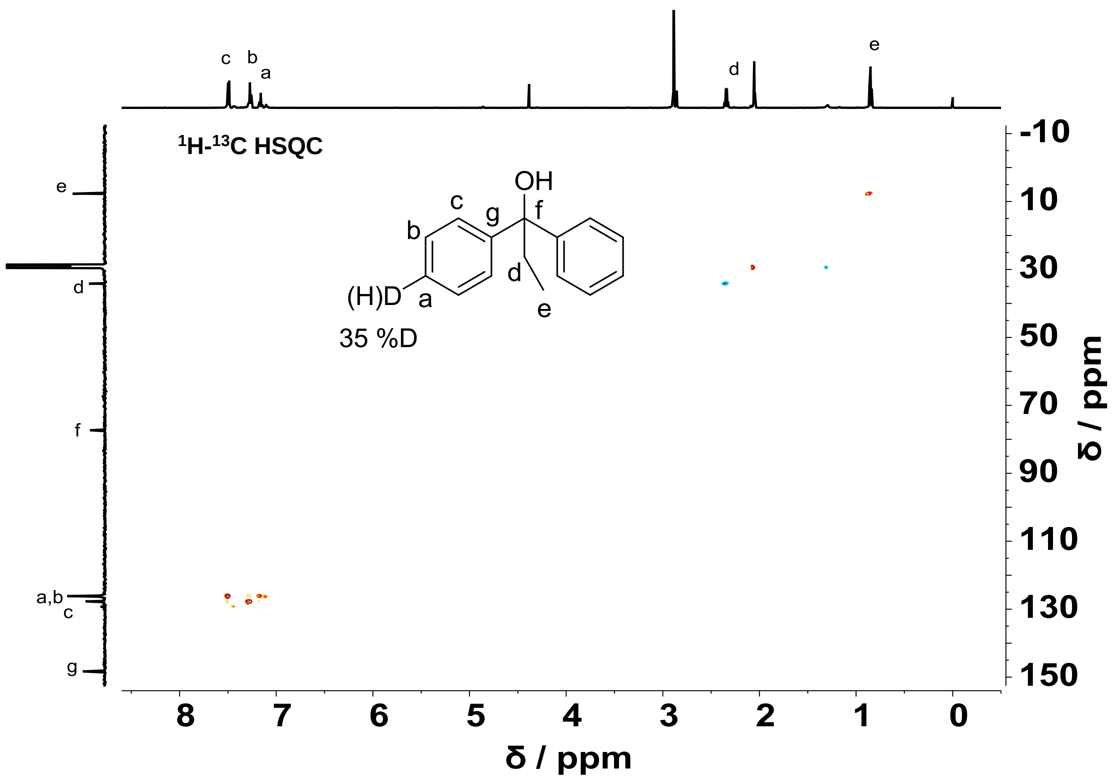

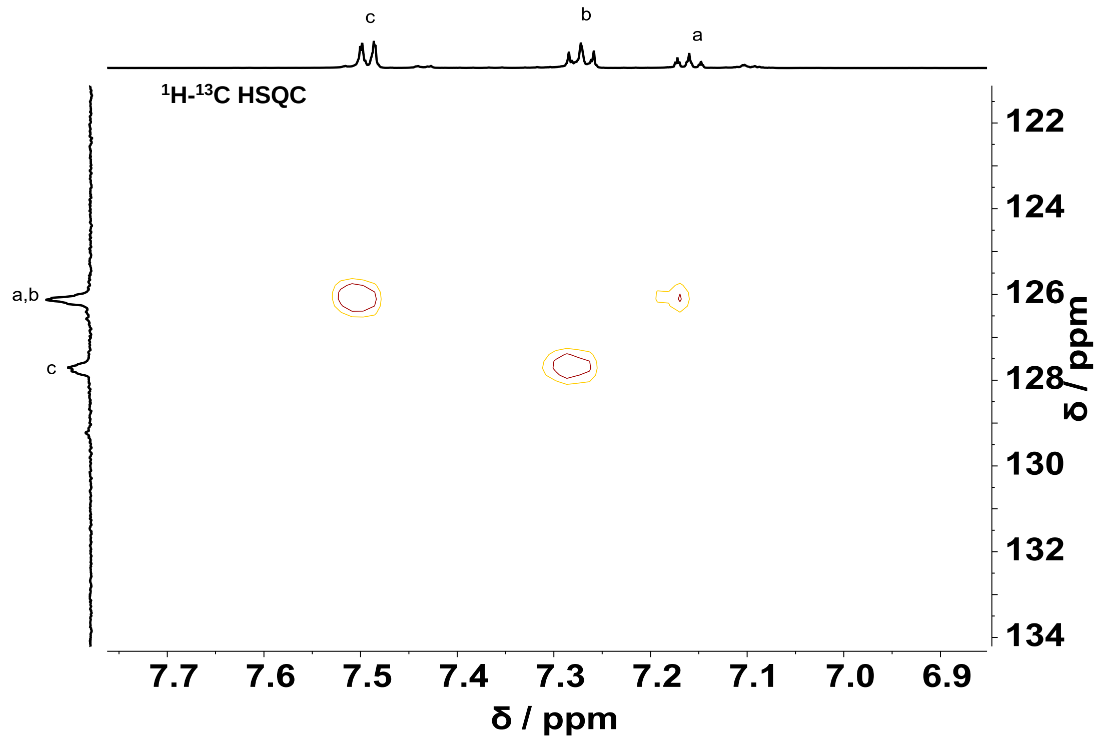

Figure S31. ^{1}H-^{13}C HSQC NMR spectra of para-deuterated diphenylethylmethanol, related to **Figure 3.**

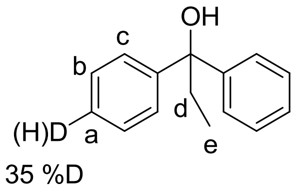

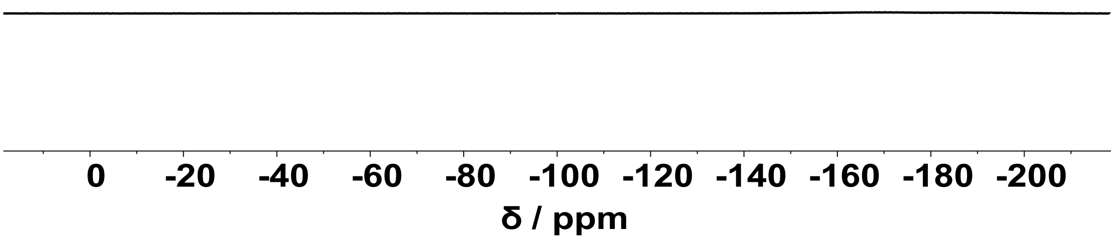

Figure S32. ^{19}F NMR spectrum of para-deuterated diphenylethylmethanol, related to **Figure 3.**

Supplemental Tables

Table S1. The influence of catalysts, additives and solvents on Mg-mediated C-F activation, related to **Figure 1.**

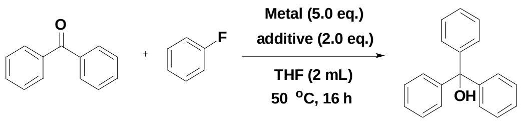

Entry	Metal	Additive (X eq.)	Solvent(2 mL)	Yield /% [a]
1	Mg	LiCl (2.0 eq.)	THF	15
2	In	LiCl (2.0 eq.)	THF	Trace
3	Zn	LiCl (2.0 eq.)	THF	Trace
4	Mn	LiCl (2.0 eq.)	THF	Trace
5	--	LiCl (2.0 eq.)	THF	Trace
6	Mg	LiCl (2.0 eq.)	Toluene	Trace
7	Mg	LiCl (2.0 eq.)	Dioxane	Trace
8	Mg	LiCl (2.0 eq.)	DME	Trace
9	Mg	LiCl (2.0 eq.)	CH_3CN	Trace
10	Mg	LiCl (2.0 eq.)	Ether	Trace
11	Mg	LiCl (2.0 eq.)	Tert-Butyl methyl ether (MTBE)	Trace
12	Mg	LiCl (2.0 eq.)	Isopropyl ether	Trace
13	Mg	$BrCH_2CH_2Br$ (0.2 eq.)	THF	25
14	Mg	$BrCH_2CH_2Br$ (2.0 eq.)	THF	6
15	Mg	NaI (2.0 eq.)	THF	Trace
16	Mg	LiI (2.0 eq.)	THF	Trace
17	Mg	$InCl_3$ (2.0 eq.)	THF	Trace
18	Mg	NaCl (2.0 eq.)	THF	Trace
19	Mg	$SnCl_2 . 2H_2O$ (2.0 eq.)	THF	Trace
20	Mg	$LiClO_4$ (2.0 eq.)	THF	Trace
21	Mg	$AlCl_3$ (2.0 eq.)	THF	Trace
22	Mg	$ZnCl_2$ (2.0 eq.)	THF	Trace
23	Mg	$FeCl_3$ (2.0 eq.)	THF	Trace
24	Mg	$TiCl_4$ (2.0 eq.)	THF	Trace
25	Mg	$RhCl_3$ (2.0 eq.)	THF	Trace
26	Mg	$HgCl_2$ (2.0 eq.)	THF	Trace
27	Mg	$PbCl_2$ (2.0 eq.)	THF	Trace
28	Mg	$NiCl_2$ (2.0 eq.)	THF	Trace
29	Mg	CuI (2.0 eq.)	THF	Trace
30	Mg	$CeCl_3$ (2.0 eq.)	THF	Trace
31	Mg	$TiCl_4$ (2.0 eq.)	THF	Trace
32	Mg	KI (2.0 eq.)	THF	Trace
33	Mg	KBr (2.0 eq.)	THF	Trace
34	Mg	LiCl (0.2 eq.)	THF	Trace

[a] isolated yield.

Table S2. Reactivity of fluorobenzene with different substrates in the Barbier reaction (A) and the Grignard reaction (B), related to **Figure 1.**

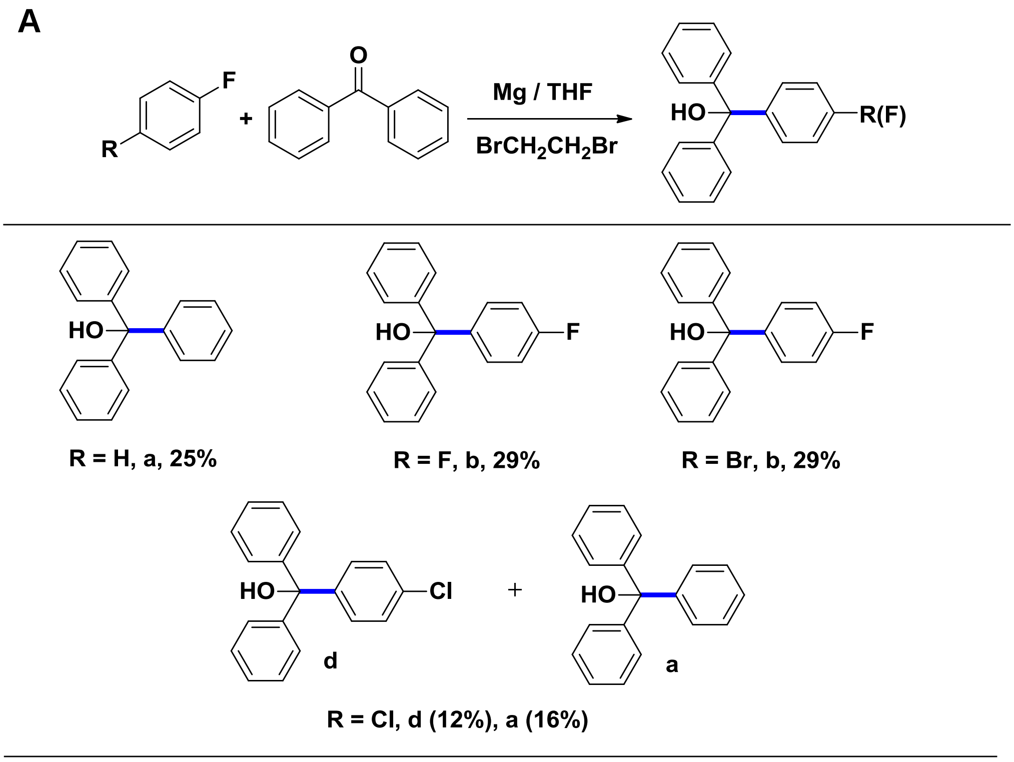

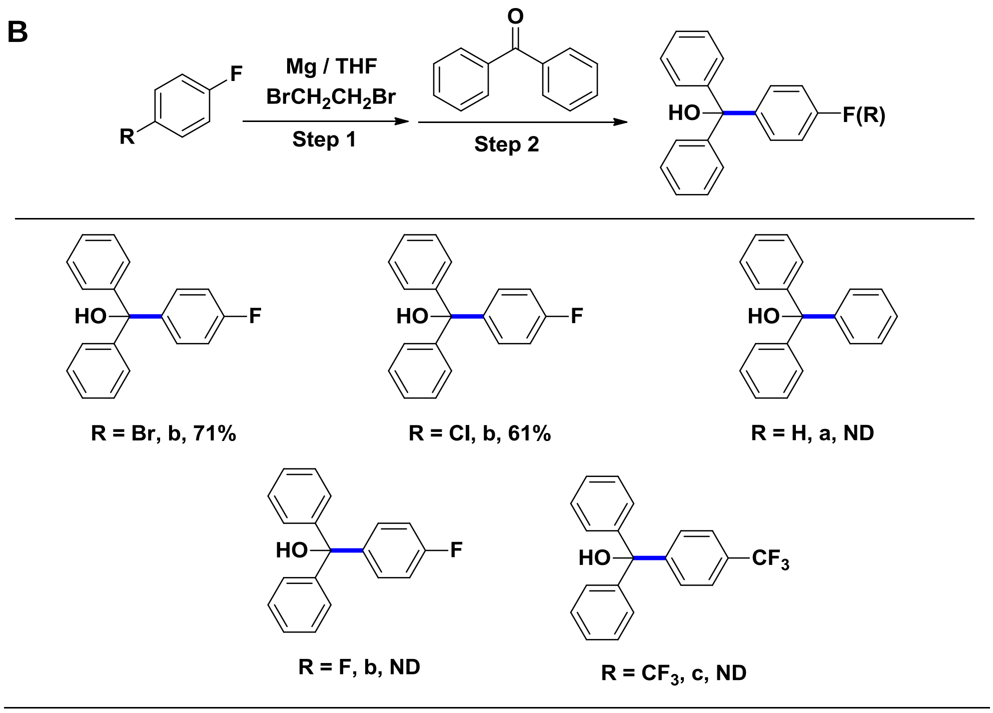

Table S3. Reactivity of C-F, C-Cl and C-Br in Barbier reaction and Grignard reaction, related to **Figure 1.**

Entry	Reactant	Reaction Type	Product and Yield
1	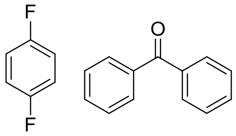	**Barbier reaction**	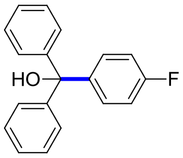 **29%**
		**Grignard reaction**	— —
2	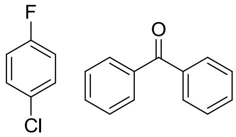	**Barbier reaction**	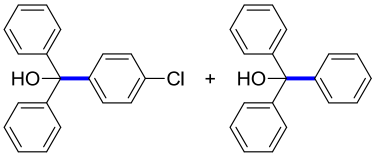 **12%** **16%**
		**Grignard reaction**	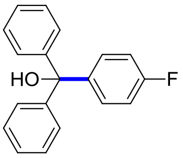 **61%**
3	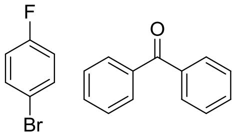	**Barbier reaction**	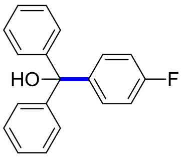 **29%**
		**Grignard reaction**	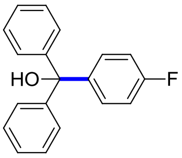 **71%**

Table S4. Monomer structures and results of polymers synthesized via self-condensing ketyl polymerization, related to **Figure 4.**

Entry	Monomer	Polymer	Conversion(%)[a]	Yield(%)	M_n[b]	*Đ*	PIE[c]
1	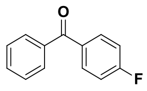	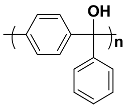	89.9	70.0	3400	1.40	AIE
2	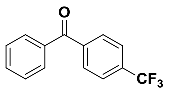	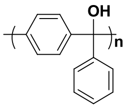	92.5	71.4	4900	1.38	AIE
3	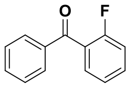	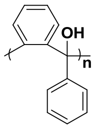	86.2	62.5	3800	1.25	ACQ
4	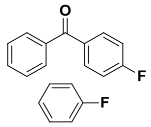	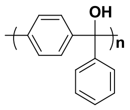	88.5	68.8	3300	1.38	AIE
5	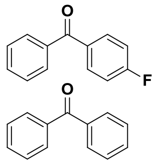	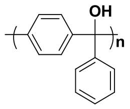	N/A	N/A	N/A	N/A	N/A
6	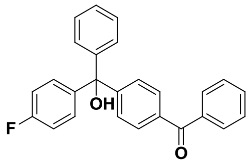	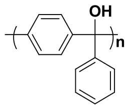	N/A	N/A	N/A	N/A	N/A
7	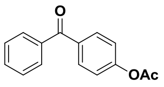	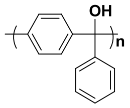	N/A	N/A	N/A	N/A	N/A
8	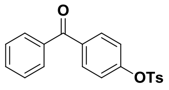	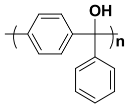	N/A	N/A	N/A	N/A	N/A
9	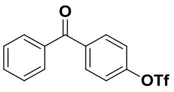	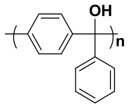	N/A	N/A	N/A	N/A	N/A

[a] calculated from ^{1}H NMR spectroscopy by comparing the integral ratio between terminal group and polymer;

[b] measured by GPC;

[c] polymerization-induced emission.

Transparent Methods

Materials

All chemicals and reagents were purchased from commercial suppliers and used without further purification. THF was distilled from Na/benzophenone prior to use.

Characterization methods

Nuclear magnetic resonance (NMR) spectroscopy. The NMR spectroscopy was performed on Bruker Ascend TM 400 or ECZ600S spectrometer, which $CDCl_3$ was used as solvent and tetramethylsilane as an internal standard. Respectively, ^1H NMR measured at concentration of 10-20 mg/mL, ^{13}C NMR at concentration of 60-80 mg/mL and ^{19}F NMR at concentration of 40-60 mg/mL.

Gel permeation chromatography (GPC). GPC curves of F-PTPMs were performed on a Viscotek TDA 302 triple detector array equipped with one TSK-Gel GMHHR-N column, which aimed at determined the molecular weight and molecular weight distribution. In detail, THF was used as eluent at a flow rate of 1.0 mL/min and carried out at 30 ^{o}C. Monodispersed polystyrene standards were used in the calibration of molecular weight and molecular weight distribution.

Fourier transform infrared (FT-IR) spectroscopy. FT-IR spectra were performed on a TENSOR II FT-IR Spectrometre (Bruker, Germany). The spectra were collected at room temperature and recorded from an accumulation of 16 scans in the range of 4000-400 cm^{-1}. The OPUS v7.5 software auto-corrected the spectral base line and calculated the second derivative spectra.

Differential scanning calorimeter (DSC) curve. Thermal characterization was performed on a DSC214 polyma (NETZSCH, Germany). The test atmosphere and purge gas were high pure N_2, and the flow rate of N_2 was 60 mL/min. An empty Al crucible was used as the reference. The temperature range of test was 25-200°C with a heating rate of 10 K/min, and the cooling rate was 10 K/min. After removing the thermal history of the material by raise and cool down twice, the thermodynamic curves were measured.

MALDI-TOF mass spectrometry. Mass spectrum was recorded on a Microflex MALDI-TOF mass spectrometer (Bruker Daltonics) in the positive-ion mode.

High-resolution mass spectrometry. HRMS data were recorded on Waters Micromass GCT Premier.

UV-vis absorption. Sample transmittance recorded on a Shimadzu UV-2450 UV-Vis spectrophotometer at room temperature in THF/Water mixtures with different water fractions (f_w, vol%).

Photophysical properties. The measurement of the polymers in solvent/nonsolvent mixtures were tested. In this study, THF was selected as good solvent for polymers and water as aggregation-inducing nonsolvent. The fluorescence spectra of polymers in THF/Water mixtures with different water content were recorded respectively on an Edinburgh Instruments FLS5 fluorescence spectrofluorometer and Shimadzu Instruments RF-5301PC fluorescence spectrophotometer.

Theoretical calculation. The hybrid density functional theory B3LYP with Grimme's dispersion correction (DFT-D3) was employed for all geometrical optimizations, thermal energy calculations and frequency analyses in gas phase combined with basis sets Def2-SVP implemented in the Gaussian 09 package for all atoms.

Reaction Procedures

General synthetic procedure for benzophenones with different leaving group

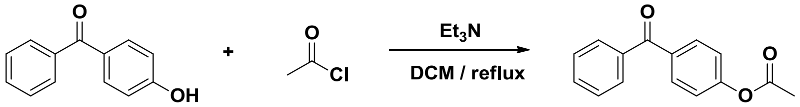

Synthesis of 4-acetoxybenzophenone. Under nitrogen protection, a solution of 4-hydroxybenzophenone (2.42 g, 12 mmol), acetylchloride (1.28 mL, 18 mmol) and triethylamine (3.36 mL, 24 mmol) in dichloromethane (150 mL) was heated under reflux for 20 h. After cooling down to room temperature, the mixture was workup with H_2O for three times. The organic phase was dried over $MgSO_4$ and concentrated under reduced pressure and the residue was purified by column chromatography on silica gel (PE/EA =20/1-3/1) to give a white solid (2.19 g, 76%). ^{1}H NMR (400 MHz, $CDCl_3$): δ = 7.84–7.87 (2H, m), 7.79–7.81 (2H, m), 7.58–7.61 (1H, m), 7.47–7.50 (2H, m), 7.20–7.24(2H, m), 2.34 (3H, s); ^{13}C NMR (101 MHz, $CDCl_3$): δ =195.6, 169.1, 154.0, 137.5, 135.2, 132.5, 131.3, 130.1, 128.5, 121.6, 21.3.

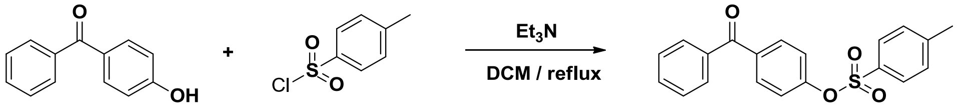

Synthesis of 4-benzoylphenyl tosylate. Under nitrogen protection, a solution of 4-hydroxybenzophenone (2.42 g, 12 mmol), p-toluenesulfonyl chloride (3.558g, 18 mmol) and triethylamine (3.36 mL, 24 mmol) in dichloromethane (150 mL) was heated under reflux for 20 h. After cooling down to room temperature, the mixture was workup with H_2O for three times. The organic phase was dried over $MgSO_4$ and concentrated under reduced pressure and the residue was purified by column chromatography on silica gel (PE/EA =20/1-3/1) to give a white solid (3.38 g, 80%). ^{1}H NMR (400 MHz, $CDCl_3$): δ = 7.81-7.70 (6H, t), 7.60-7.55 (1H, t), 7.50-7.43 (2H, t), 7.39–7.29 (2H, d), 7.13–7.09 (2H, d), 2.43 (3H, s); ^{13}C NMR (101 MHz, $CDCl_3$): δ =195.31, 152.61, 146.01, 137.16, 136.28, 132.89, 131.86, 130.13, 128.51, 122.41, 21.8.

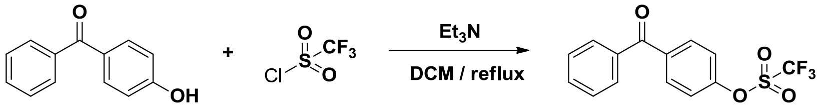

Synthesis of 4-benzoylphenyl triflate. Under nitrogen protection, a solution of 4-hydroxybenzophenone (1.12 g, 6.0 mmol), trifluoromathanesulfonyl chloride (1.52 g, 9.0 mmol) and triethylamine (1.68 mL, 12 mmol) in dichloromethane (70 mL) was heated under reflux for 20 h. After cooling down to room temperature, the mixture was workup with H_2O for three times. The organic phase was dried over $MgSO_4$ and concentrated under reduced pressure and the residue was purified by column chromatography on silica gel (PE/EA =20/1-3/1) to give a white solid (1.41 g, 72%). ^{1}H NMR (400 MHz, $CDCl_3$): δ = 7.94-7.89 (2H, d), 7.82-7.77 (2H, d), 7.68-7.60 (1H, t), 7.55-7.48 (2H, t), 7.45-7.37 (2H, d); ^{13}C NMR (101 MHz, $CDCl_3$): δ =195.14, 152.10, 137.62, 136.51, 133.36, 132.25, 130.26, 128.47, 121.55; ^{19}F NMR (376 MHz, $CDCl_3$): δ=-72.35.

General synthetic procedure for self-condensing ketyl radical anion polymerization

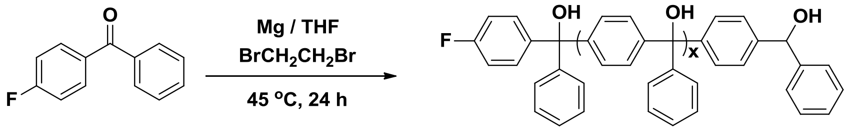

Synthesis of fluoro-PTPM with 4-fluorobenzophenone. Under nitrogen protection, freshly peeled Mg scraps 0.144 g (6.0 mmol) was added into flame-dried Schlenk tube, then the solution of monomer which 4-fluorobenzophenone 1.0 g (5.0 mmol) dissolved in THF (10 mL) was injected into the tube with a syringe. After stirring for 10 min, 0.2 eq. 1, 2-dibromoethane was added to the Schlenk tube, and then reacted at 45 °C for 24 h. The reaction was quenched and hydrolysis by saturated aqueous ammonium chloride at room temperature, then workup with dichloromethane/ water. The organic solution was dried with anhydrous $MgSO_4$ and concentrated under reduced pressure. The oily liquids product was diluted with dichloromethane then precipitated with excessive petroleum ether for three times, after filtered and dried under vacuum, polymer was obtained as an off-white powder (yield: 70%). ^{1}H NMR (400 MHz, $CDCl_3$): δ=7.60-6.75 (broad, -phenyl), δ=3.83-3.35 (broad, -OH); ^{13}C NMR (101 MHz, $CDCl_3$): δ=162.59, 160.66, 146.57, 145.18, 144.05-142.34, 140.18, 130.50-129.90, 128.79-125.22, 115.00-113.62, 82.84, 81.44; ^{19}F NMR (376 MHz, $CDCl_3$): δ=(-115.32)-(-116.01); $M_{n,GPC}$=3400 g/mol, $Đ$=1.40; FT-IR (KBr pellets): Wavenumber=3670-3125 (O-H), 3120-3000 (Ar-H), 1620-1465 (phenyl), 1295-1250 (Ar-F), 1090-940 (C-O).

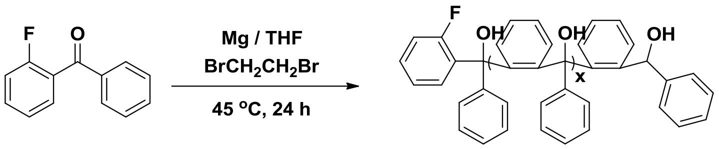

Synthesis of fluoro-PTPM with 2-fluorobenzophenone. Under nitrogen protection, freshly peeled Mg scraps 0.144 g (6.0 mmol) was added into flame-dried Schlenk tube, then the solution of 4-fluorobenzophenone 1.0 g (5.0 mmol) dissolved in THF (10 mL) was injected into the tube with a syringe. After stirring for 10 min, 0.2 eq. 1, 2-dibromoethane was added to the Schlenk tube, and then reacted at 45 °C for 24 h. The reaction was quenched and hydrolysis by saturated aqueous ammonium chloride at room temperature, then workup with dichloromethane/ water. The organic solution was dried with anhydrous $MgSO_4$ and concentrated under reduced pressure. The oily liquids product was diluted with dichloromethane then precipitated with excessive petroleum ether for three times, after filtered and dried under vacuum, polymer was obtained as an off-white powder (yield: 70%). ^{1}H NMR (400 MHz, $CDCl_3$): δ=7.85-6.64 (broad, -phenyl), δ=2.95-2.80 (broad, -OH); ^{13}C NMR (101 MHz, $CDCl_3$): δ=145.51, 140.85, 129.15-125.34, 74.72; $M_{n,GPC}$=3800 g/mol, $Đ$=1.25; FT-IR (KBr pellets): Wavenumber=3710-3120 (O-H), 3100-3000 (Ar-H), 1630-1460 (phenyl), 1295-1250 (Ar-F), 1080-950 (C-O).

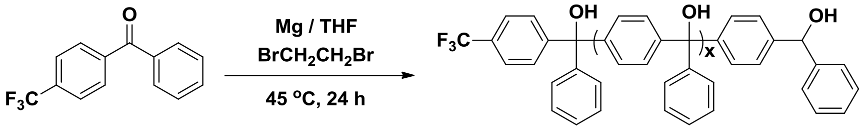

Synthesis of trifluoromethyl-PTPM with 4-trifluoromethylbenzophenone. Under nitrogen protection, freshly peeled Mg scraps 0.115 g (4.8 mmol) was added into flame-dried Schlenk tube, then the solution of 4-trifluoromethylbenzophenone 1.0 g (4.0 mmol) dissolved in THF (8 mL) was injected into the tube with a syringe. After stirring for 10 min, 0.2 eq. 1, 2-dibromoethane was added to the Schlenk tube, and then reacted at 45 °C for 24 h. The reaction was quenched and hydrolysis by saturated aqueous ammonium chloride at room temperature, then workup with dichloromethane/ water. The organic solution was dried with anhydrous $MgSO_4$ and concentrated under reduced pressure. The oily liquids product was diluted with dichloromethane then precipitated with excessive petroleum ether

for three times, after filtered and dried under vacuum, polymer was obtained as an off-white powder (yield: 74%). ^{1}H NMR (400 MHz, $CDCl_3$): δ=7.82-6.73 (broad, -phenyl), δ=3.38-2.85 (broad, -OH); ^{13}C NMR (101 MHz, $CDCl_3$): δ=145.25, 137.52, 13.5.63, 133.41-125.36, 83.4; ^{19}F NMR (376 MHz, $CDCl_3$): δ=-65.32; $M_{n,GPC}$=4900 g/mol, Đ=1.38; FT-IR (KBr pellets): Wavenumber=3622-3128 (O-H), 3120-3000 (Ar-H), 1755-1525 (phenyl), 1045-958 (C-O).

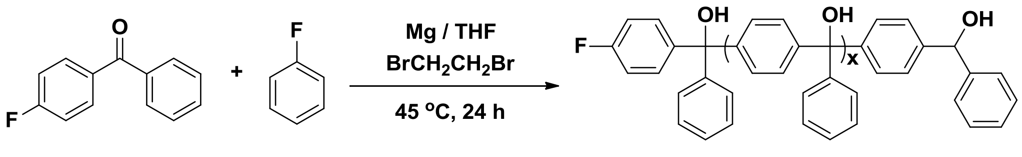

Synthesis of fluoro-PTPM with 4-fluorobenzophenone and fluorobenzene. Under nitrogen protection, freshly peeled Mg scraps 0.144 g (6.0 mmol) was added into flame-dried Schlenk tube, then the solution of monomer which 4-fluorobenzophenone 1.0 g (5.0 mmol) and fluorobenzene 0.48 g (5.0 mmol) dissolved in THF (10 mL) was injected into the tube with a syringe. After stirring for 10 min, 0.2 eq. 1, 2-dibromoethane was added to the Schlenk tube, and then reacted at 45 °C for 24 h. The reaction was quenched and hydrolysis by saturated aqueous ammonium chloride at room temperature, then workup with dichloromethane/ water. The organic solution was dried with anhydrous $MgSO_4$ and concentrated under reduced pressure. The oily liquids product was diluted with dichloromethane then precipitated with excessive petroleum ether for three times, after filtered and dried under vacuum, polymer was obtained as an off-white powder (yield: 68%). ^{1}H NMR (400 MHz, $CDCl_3$): δ=7.60-6.75 (broad, -phenyl), δ=3.83-3.35 (broad, -OH); ^{13}C NMR (101 MHz, $CDCl_3$): δ=162.59, 160.66, 146.57, 145.18, 144.05-142.34, 140.18, 130.50-129.90, 128.79-125.22, 115.00-113.62, 82.84, 81.44; ^{19}F NMR (376 MHz, $CDCl_3$): δ=(-115.32)-(-116.01); $M_{n,GPC}$=3300 g/mol, Đ=1.38; FT-IR (KBr pellets): Wavenumber=3670-3125 (O-H), 3120-3000 (Ar-H), 1620-1465 (phenyl), 1295-1250 (Ar-F), 1090-940 (C-O).

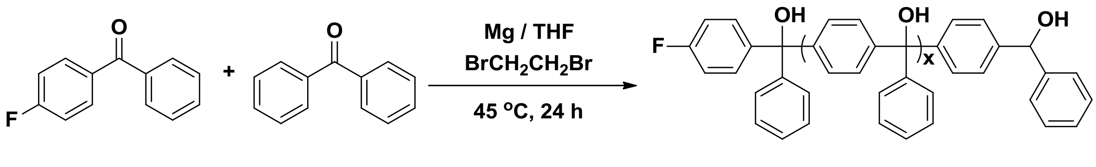

Synthesis of fluoro-PTPM with 4-fluorobenzophenone and benzophenone. Under nitrogen protection, freshly peeled Mg scraps 0.144 g (6.0 mmol) was added into flame-dried Schlenk tube, then the solution of monomer which 4-fluorobenzophenone 1.0 g (5.0 mmol) and benzophenone 0.91 g (5.0 mmol) dissolved in THF (10 mL) was injected into the tube with a syringe. After stirring for 10 min, 0.2 eq. 1, 2-dibromoethane was added to the Schlenk tube, and then reacted at 45 ^{o}C for 24 h. The reaction was quenched and hydrolysis by saturated aqueous ammonium chloride at room temperature, then workup with dichloromethane/ water. The organic solution was dried with anhydrous $MgSO_4$ and concentrated under reduced pressure. Product can't be precipitated in petroleum ether and only trace polymer detected by NMR.

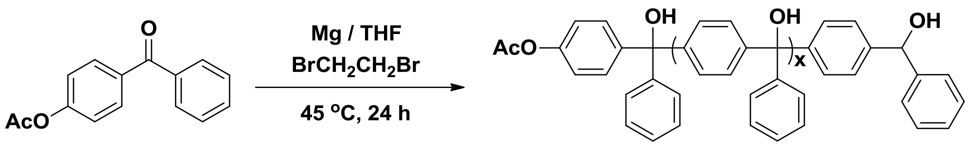

Synthesis of acetoxy-PTPM with 4-acetoxybenzophenone. Under nitrogen protection, freshly peeled Mg scraps 0.144 g (6.0 mmol) was added into flame-dried Schlenk tube, then the solution of monomer which 4-acetoxybenzophenone 1.2 g (5.0 mmol) dissolved in THF (10 mL) was injected into the tube with a syringe. After stirring for 10 min, 0.2 eq. 1, 2-dibromoethane was added to the Schlenk tube, and then reacted at 45 ^{o}C for 24 h. The reaction was quenched and hydrolysis by saturated aqueous ammonium chloride at room temperature, then workup with dichloromethane/ water. The organic solution was dried with anhydrous $MgSO_4$ and concentrated under reduced pressure. Product can't be precipitated in petroleum ether and no polymer detected by NMR.

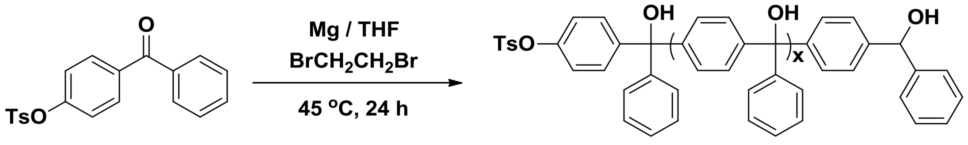

Synthesis of tosylate-PTPM with 4-benzoylphenyl tosylate. Under nitrogen protection, freshly peeled Mg scraps 0.144 g (6.0 mmol) was added into flame-dried Schlenk tube, then the solution of monomer which 4-benzoylphenyl tosylate 1.8 g (5.0 mmol) dissolved in THF (10 mL) was injected

into the tube with a syringe. After stirring for 10 min, 0.2 eq. 1, 2-dibromoethane was added to the Schlenk tube, and then reacted at 45 °C for 24 h. The reaction was quenched and hydrolysis by saturated aqueous ammonium chloride at room temperature, then workup with dichloromethane/ water. The organic solution was dried with anhydrous $MgSO_4$ and concentrated under reduced pressure. Product can’t be precipitated in petroleum ether and no polymer detected by NMR.

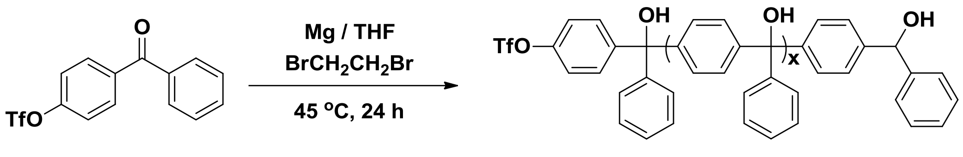

Synthesis of triflate-PTPM with 4-benzoylphenyl triflate. Under nitrogen protection, freshly peeled Mg scraps 0.144 g (6.0 mmol) was added into flame-dried Schlenk tube, then the solution of monomer which 4-benzoylphenyl triflate 1.65 g (5.0 mmol) dissolved in THF (10 mL) was injected into the tube with a syringe. After stirring for 10 min, 0.2 eq. 1, 2-dibromoethane was added to the Schlenk tube, and then reacted at 45 °C for 24 h. The reaction was quenched and hydrolysis by saturated aqueous ammonium chloride at room temperature, then workup with dichloromethane/ water. The organic solution was dried with anhydrous $MgSO_4$ and concentrated under reduced pressure. Product can’t be precipitated in petroleum ether and no polymer detected by NMR.

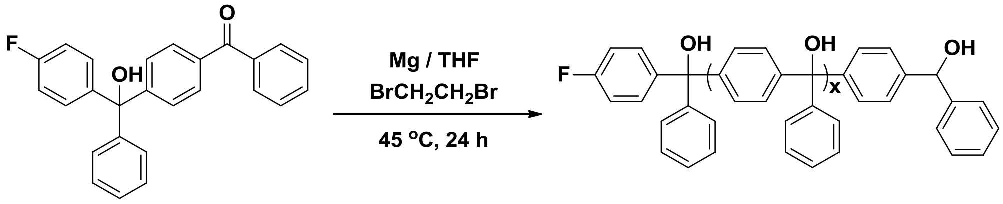

Polymerization procedure of dimer intermediate. Under nitrogen protection, freshly peeled Mg scraps 0.072 g (3.0 mmol) was added into flame-dried Schlenk tube, then the solution of monomer which dimer intermediate 0.96 g (2.5 mmol) dissolved in THF (10 mL) was injected into the tube with a syringe. After stirring for 10 min, 0.2 eq. 1, 2-dibromoethane was added to the Schlenk tube, and then reacted at 45 °C for 24 h. The reaction was quenched and hydrolysis by saturated aqueous ammonium chloride at room temperature, then workup with dichloromethane/ water. The organic solution was dried with anhydrous $MgSO_4$ and concentrated under reduced pressure. Product can’t precipitated in

petroleum ether and no polymer detected by NMR.

General synthetic procedure for the Barbier C-F activation reaction

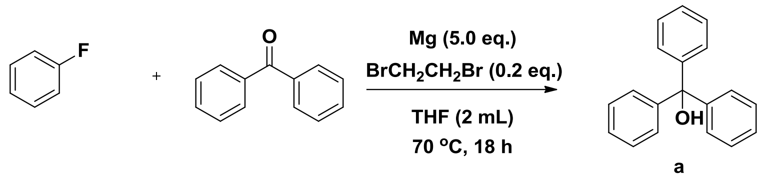

Barbier C-F Activation Reaction with fluorobenzene and benzophenone. Under nitrogen protection, freshly peeled Mg scraps 0.120 g (5.0 mmol) was added into flame-dried Schlenk tube, then the solution of reactant and additives which benzophenone 0.364 g (2.0 mmol), fluorobenzene 0.096 g (1.0 mmol) and 20 μL of 1, 2-dibromoethane dissolved in THF (2 mL) was injected into the tube with a syringe. After the reaction was refluxed for 18 h, the solution was quenched and hydrolysis by 20 mL saturated aqueous ammonium chloride at room temperature, then workup with dichloromethane/water. The organic solution was dried with anhydrous $MgSO_4$ and concentrated under reduced pressure and the residue was purified by column chromatography on silica gel (PE/EA =20/1-3/1) to give 25% yield of a as a white solid. ^{1}H NMR (400 MHz, $CDCl_3$): δ 7.38–7.17 (m, 15H), 2.82 (s, 1H). ^{13}C NMR (101 MHz, $CDCl_3$): 146.87, 127.98, 127.96, 127.31, 82.06, 77.39, 77.08, 76.76.

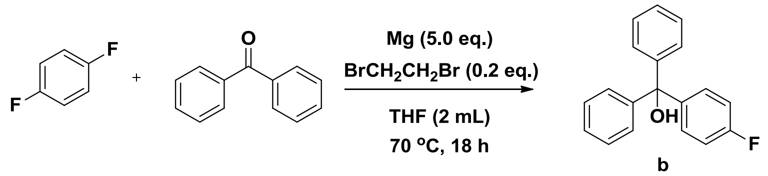

Barbier C-F Activation Reaction with 1,4-difluorobenzene and benzophenone. Under nitrogen protection, freshly peeled Mg scraps 0.120 g (5.0 mmol) was added into flame-dried Schlenk tube, then the solution of reactant and additives which benzophenone 0.364 g (2.0 mmol), 1,4-difluorobenzene 0.114 g (1.0 mmol) and 20 μL of 1, 2-dibromoethane dissolved in THF (2 mL) was injected into the tube with a syringe. After the reaction was refluxed for 18 h, the solution was quenched and hydrolysis by 20 mL saturated aqueous ammonium chloride at room temperature, then workup with

dichloromethane/water. The organic solution was dried with anhydrous $MgSO_4$ and concentrated under reduced pressure and the residue was purified by column chromatography on silica gel (PE/EA =20/1-3/1) to give 29% yield of b as a white solid. ^1H NMR (400 MHz, $CDCl_3$): δ 7.39–7.22 (m, 12H), 6.98 (d, J = 8.6 Hz, 2H), 2.82 (s, 1H). ^{13}C NMR (101 MHz, $CDCl_3$): δ 163.16, 160.71, 146.73, 142.71, 129.79, 129.71, 128.64, 128.31, 128.14, 128.06, 127.85, 127.45, 127.32, 126.98, 126.78, 126.14, 126.02, 114.81, 114.60, 81.70. ^{19}F NMR (376 MHz, $CDCl_3$) δ -115.52.

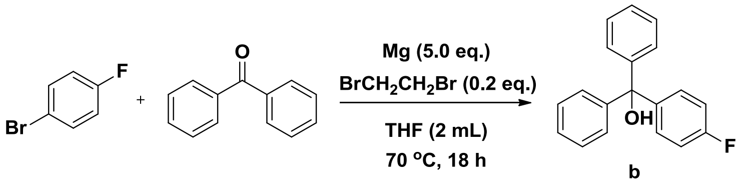

Barbier C-F activation reaction with 4-bromofluorobenzene and benzophenone. Under nitrogen protection, freshly peeled Mg scraps 0.120 g (5.0 mmol) was added into flame-dried Schlenk tube, then the solution of reactant and additives which benzophenone 0.364 g (2.0 mmol), 4-bromofluorobenzene 0.175 g (1.0 mmol) and 20 μL of 1, 2-dibromoethane dissolved in THF (2 mL) was injected into the tube with a syringe. After the reaction was refluxed for 18 h, the solution was quenched and hydrolysis by 20 mL saturated aqueous ammonium chloride at room temperature, then workup with dichloromethane/water. The organic solution was dried with anhydrous $MgSO_4$ and concentrated under reduced pressure and the residue was purified by column chromatography on silica gel (PE/EA =20/1-3/1) to give 36% yield of b as a white solid. ^1H NMR (400 MHz, $CDCl_3$): δ 7.39–7.22 (m, 12H), 6.98 (d, J = 8.6 Hz, 2H), 2.82 (s, 1H). ^{13}C NMR (101 MHz, $CDCl_3$): δ 163.16, 160.71, 146.73, 142.71, 129.79, 129.71, 128.64, 128.31, 128.14, 128.06, 127.85, 127.45, 127.32, 126.98, 126.78, 126.14, 126.02, 114.81, 114.60, 81.70. ^{19}F NMR (376 MHz, $CDCl_3$): δ -115.52.

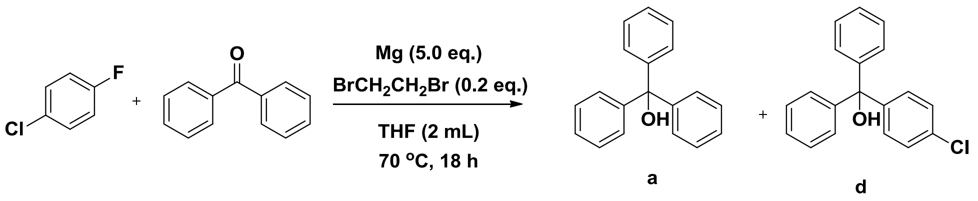

Barbier C-F activation reaction with 4-chlorofluorobenzene and benzophenone. Under nitrogen protection, freshly peeled Mg scraps 0.120 g (5.0 mmol) was added into flame-dried Schlenk tube, then the solution of reactant and additives which benzophenone 0.364 g (2.0 mmol), 4-chlorofluorobenzene 0.130 g (1.0 mmol) and 20 μL of 1, 2-dibromoethane dissolved in THF (2 mL) was injected into the tube with a syringe. After the reaction was refluxed for 18 h, the solution was quenched and hydrolysis by 20 mL saturated aqueous ammonium chloride at room temperature, then workup with dichloromethane/water. The organic solution was dried with anhydrous $MgSO_4$ and concentrated under reduced pressure and the residue was purified by column chromatography on silica gel (PE/EA =20/1-3/1) to give mixture of a and d as a white solid (a/d=1.7:2.3, determined by ^{1}H NMR, a=12% yield, d=16% yield). 1H NMR (400 MHz, $CDCl_3$): δ 7.33–7.12 (m, 42H), 3.03 (s, 1.09H), 2.83 (s, 1H), 2.81 (s, 1.25H). ^{13}C NMR (101 MHz, $CDCl_3$): δ 146.86, 146.46, 145.36, 144.16, 133.17, 129.38, 128.62, 128.10, 128.05, 127.95, 127.83, 127.52, 127.31, 127.28, 126.97, 126.13, 83.04, 82.05, 81.69.

General synthetic procedure for the Grignard reaction with fluoro-compounds

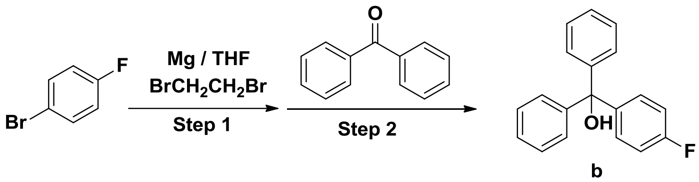

Grignard reaction with 4-bromofluorobenzene and benzophenone. Under nitrogen protection, freshly peeled Mg scraps 0.120 g (5.0 mmol) was added into flame-dried Schlenk tube, then the solution of fluoro-compounds which 4-bromofluorobenzene 0.175 g (1.0 mmol) dissolved in THF (2 mL) was injected into the tube with a syringe. After stirring for 10 min, 0.1 mL 1, 2-dibromoethane was added to the Schlenk tube, then refluxed for 2 h. Then the reaction solution was injected to benzophenone 0.364 g (2.0 mmol) which dissolved in THF (2 mL). After stirring for 24 h at room

temperature, the reaction solution was quenched and hydrolysis by 20 mL saturated aqueous ammonium chloride, then workup with dichloromethane/water. The organic solution was dried with anhydrous $MgSO_4$ and concentrated under reduced pressure and the residue was purified by column chromatography on silica gel (PE/EA =20/1-3/1) to give 71% yield of b as a white solid. ^1H NMR (400 MHz, $CDCl_3$): δ 7.39–7.22 (m, 12H), 6.98 (d, J = 8.6 Hz, 2H), 2.82 (s, 1H). ^{13}C NMR (101 MHz, $CDCl_3$): δ 163.16, 160.71, 146.73, 142.71, 129.79, 129.71, 128.64, 128.31, 128.14, 128.06, 127.85, 127.45, 127.32, 126.98, 126.78, 126.14, 126.02, 114.81, 114.60, 81.70. ^{19}F NMR (376 MHz, $CDCl_3$) δ -115.52.

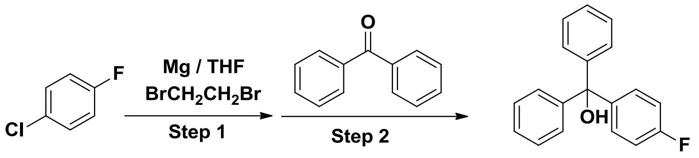

b

Grignard reaction with 4-chlorofluorobenzene and benzophenone. Under nitrogen protection, freshly peeled Mg scraps 0.120 g (5.0 mmol) was added into flame-dried Schlenk tube, then the solution of fluoro-compounds which 4-chlorofluorobenzene 0.130 g (1.0 mmol) dissolved in THF (2 mL) was injected into the tube with a syringe. After stirring for 10 min, 0.1 mL 1, 2-dibromoethane was added to the Schlenk tube, then refluxed for 2 h. Then the reaction solution was injected to benzophenone 0.364 g (2.0 mmol) which dissolved in THF (2 mL). After stirring for 24 h at room temperature, the reaction solution was quenched and hydrolysis by 20 mL saturated aqueous ammonium chloride, then workup with dichloromethane/water. The organic solution was dried with anhydrous $MgSO_4$ and concentrated under reduced pressure and the residue was purified by column chromatography on silica gel (PE/EA =20/1-3/1) to give 61% yield of b as a white solid. ^1H NMR (400 MHz, $CDCl_3$): δ 7.39–7.22 (m, 12H), 6.98 (d, J = 8.6 Hz, 2H), 2.82 (s, 1H). ^{13}C NMR (101 MHz, $CDCl_3$): δ 163.16, 160.71, 146.73, 142.71, 129.79, 129.71, 128.64, 128.31, 128.14, 128.06, 127.85, 127.45, 127.32, 126.98, 126.78, 126.14, 126.02, 114.81, 114.60, 81.70. ^{19}F NMR (376 MHz, $CDCl_3$) δ -115.52.

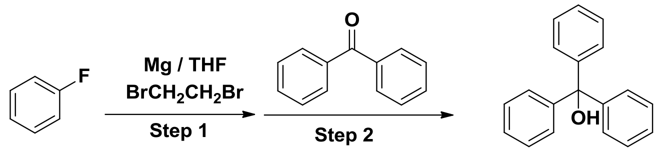

a

Grignard reaction with fluorobenzene and benzophenone. Under nitrogen protection, freshly peeled Mg scraps 0.120 g (5.0 mmol) was added into flame-dried Schlenk tube, then the solution of fluoro-compounds which fluorobenzene 0.096 g (1.0 mmol) dissolved in THF (2 mL) was injected into the tube with a syringe. After stirring for 10 min, 0.1 mL 1, 2-dibromoethane was added to the Schlenk tube, then refluxed for 2 h. Then the reaction solution was injected to benzophenone 0.364 g (2.0 mmol) which dissolved in THF (2 mL). After stirring for 24 h at room temperature, the reaction solution was quenched and hydrolysis by 20 mL saturated aqueous ammonium chloride, then workup with dichloromethane/water. The organic solution was dried with anhydrous $MgSO_4$ and concentrated under reduced pressure. The product was not detected by TLC and GC-MS.

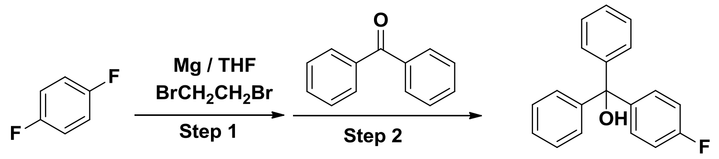

b

Grignard reaction with 1,4-difluorobenzene and benzophenone. Under nitrogen protection, freshly peeled Mg scraps 0.120 g (5.0 mmol) was added into flame-dried Schlenk tube, then the solution of fluoro-compounds which 1, 4-difluorobenzene 0.114 g (1.0 mmol) dissolved in THF (2 mL) was injected into the tube with a syringe. After stirring for 10 min, 0.1 mL 1, 2-dibromoethane was added to the Schlenk tube, then refluxed for 2 h. Then the reaction solution was injected to benzophenone 0.364 g (2.0 mmol) which dissolved in THF (2 mL). After stirring for 24 h at room temperature, the reaction solution was quenched and hydrolysis by 20 mL saturated aqueous ammonium chloride, then workup with dichloromethane/water. The organic solution was dried with anhydrous $MgSO_4$ and concentrated under reduced pressure. The product was not detected by TLC and GC-MS.

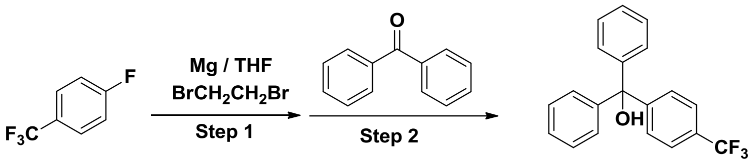

Grignard reaction with 4-fluorobenzotrifluoride and benzophenone. Under nitrogen protection, freshly peeled Mg scraps 0.120 g (5.0 mmol) was added into flame-dried Schlenk tube, then the solution of fluoro-compounds which 4-fluorobenzotrifluoride 0.114 g (1.0 mmol) dissolved in THF (2 mL) was injected into the tube with a syringe. After stirring for 10 min, 0.1 mL 1, 2-dibromoethane was added to the Schlenk tube, then refluxed for 2 h. Then the reaction solution was injected to benzophenone 0.364 g (2.0 mmol) which dissolved in THF (2 mL). After stirring for 24 h at room temperature, the reaction solution was quenched and hydrolysis by 20 mL saturated aqueous ammonium chloride, then workup with dichloromethane/water. The organic solution was dried with anhydrous $MgSO_4$ and concentrated under reduced pressure. The product was not detected by TLC and GC-MS.

General Procedure for Experimental Mechanistic Studies

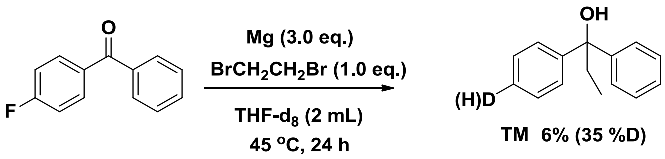

Synthesis of fluoro-PTPM with 4-fluorobenzophenone in THF-*d*$_8$. Under nitrogen protection, freshly peeled Mg scraps 0.144 g (6.0 mmol) was added into flame-dried Schlenk tube, then the solution of 4-fluorobenzophenone 0.4 g (2.0 mmol) dissolved in dry THF-d_8 (2 mL) was injected into the tube with a syringe. After stirring for 10 min, 1 eq. of 1, 2-dibromoethane was added to the Schlenk tube, and then reacted at 45 ^{o}C for 24 h. The reaction was quenched and hydrolysis by saturated aqueous ammonium chloride at room temperature, then workup with dichloromethane/water. The organic solution was dried with anhydrous $MgSO_4$ and concentrated under reduced pressure and then the residue was purified by column chromatography on silica gel (PE/EA =20/1-3/1) to give 6% yield of para-deuterated diphenylethylmethanol as a white solid. ^{1}H NMR (600 MHz, Acetone-d_6) δ 7.48–

7.45 (m, 4H), (dd, J = 8.4, 1.1 Hz, 4H), 7.26–7.21 (m, 4H), 7.14–7.10 (m, 1.65H), 4.35 (S, 1H), 2.31 (q, J = 7.3 Hz, 2H), 0.82 (t, J = 7.3 Hz, 3H). ^{13}C NMR (151 MHz, Acetone-d_6): δ 148.32, 127.71, 126.13, 77.33, 34.15, 7.67. HRMS (EI$^+$) calcd for $[C_{15}H_{15}DO]^+$ (M^+):213.1264, found: 213.1274.
